# Intratumoral microbiota is associated with prognosis in patients with adrenocortical carcinoma

**DOI:** 10.1002/imt2.102

**Published:** 2023-04-05

**Authors:** Yuqing Li, Dengwei Zhang, Minghua Wang, Haowen Jiang, Chenchen Feng, Yong‐Xin Li

**Affiliations:** ^1^ Department of Urology, Huashan Hospital Fudan University Shanghai China; ^2^ Department of Chemistry and The Swire Institute of Marine Science The University of Hong Kong Hong Kong China; ^3^ Southern Marine Science and Engineering Guangdong Laboratory (Guangzhou) Guangzhou China

**Keywords:** adrenocortical carcinoma, intratumoral microbiota, microbiome, prognosis

## Abstract

Adrenocortical carcinoma (ACC) is a rare but aggressive malignancy. Recent studies have discovered a pivotal role of the intratumoral microbiota in various cancers, yet it remains elusive in ACC. Here, we explored the intratumoral microbiome data derived from in silico identification, further validated in an in‐house cohort by bacterial 16S rRNA fluorescence in situ hybridization and lipopolysaccharide staining. Unsupervised clustering determined two naturally distinct clusters of the intratumoral microbiome in ACC, which was associated with overall survival. The incorporation of microbial signatures enhanced the prognostic performance of the clinical stage in an immunity‐dependent manner. Genetic and transcriptomic association analyses identified significant upregulation of the cell cycle and p53 signaling pathways associated with microbial signatures for worsened prognosis. Our study not only supports the presence of intratumoral bacteria but also implies a prognostic and biological role of intratumoral microbiota in ACC, which can advance a better understanding of the biology of ACC.

## Highlights

- Adrenocortical carcinoma (ACC) harbors intratumoral microbes.
- The intratumoral microbiome is associated with prognosis in ACC.
- The intratumoral microbiota might correlate with genomic events, immune status and specific carcinogenic pathways.

## Graphical abstract

The Cancer Genome Atlas primary tumors were subject to whole‐genome sequencing and RNA‐Seq, in which microbial abundance (comprising virus, archaea, and bacteria) was identified and normalized by Poore et al. (left panel). To confirm the presence of intratumor bacteria, 37 adrenocortical carcinoma tissue microarray chips from the in‐house cohort were stained for fluorescence in situ hybridization and immunohistochemistry examination. Unsupervised clustering was performed on microbiome data to explore natural clusters of patients with distinct microbiomes, thereby influencing prognoses. The microbial signatures associated with prognosis were further determined (middle panel). Defined microbial signatures were tested to improve prognosis prediction. The intratumoral microbiome was found to be associated with immune status, genetic events, and molecular pathways (right panel).

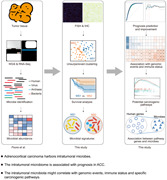

## INTRODUCTION

Adrenocortical carcinoma (ACC) is a rare but aggressive disease. Although hormone excess is present in ~50% of cases, most patients are diagnosed at an advanced stage, which confers a 5‐year survival of less than 10% [1]. As an orphan disease, the only phase III clinical trial (namely, FIRM‐ACT trial) of advanced ACC recommending an EDP+M regimen (etoposide, doxorubicin, cisplatin, and mitotane) showed a moderate effect on survival improvement [2]. A profound understanding of the biology of ACC may contribute substantially to novel treatment modalities.

Next‐generation sequencing (NGS) has enabled us to gain deeper insight into genetic and genomic alterations in ACC. To date, with NGS technologies, the Cancer Genome Atlas (TCGA) and genomic profiling by Assie et al. have depicted an unprecedented genomic understanding of this rare disease [3]. Genetic driver events include mutations in *TP53*, *CTNNB1*, *MEN1*, *PRKAR1A*, *RPL22*, *NF1*, and *MLL4*, as well as whole‐genome doubling, together with several mRNA and methylation signatures that are prognostic [4]. In addition to providing a landscape of host genetics, NGS data from tissue or blood were found to smuggle the genetic materials of microorganisms, providing an opportunity to gain a deeper understanding of the intratumoral microbiome [5].

Cancer‐resident microorganisms, especially intratumoral bacteria (ITB), have recently been revealed to play a critical role in several cancer types [5–7]. Studies on cancer‐associated microbiology have gone through three eras in general. Conventional pathology‐based studies solely identified limited carcinogenic microbiomes, such as *Helicobacter pylori* in gastric cancer and hepatitis B virus in liver cancer [8, 9]. Although limited in number, many such studies drastically updated the understanding and treatment of the disease. In the second era, which is still extending today, cancers originating from “contaminated” organs or the gastrointestinal tract were targeted [10]. The gut microbiome is now considered omnipotent in mediating various physio‐ and pathophysiological activities, including cancer [10, 11, 12, 13, 14, 15]. Metabolites of gut bacteria not only promote local carcinogenesis, but also mediate the drug sensitivity of targeted or immunotherapy of other organs [10]. Only recently have scholars been able to develop algorithms to characterize intratumoral microbes from NGS data focusing on investigating host physiology rather than resident microbes and, thus, revolutionizing our understanding of intratumoral microbiomes in various cancers [5, 6, 7]. Interrogation of the functional role of the intratumoral microbiome is of great interest, propelling onco‐microbiology into the third era.

Despite being associated with various tumors, the intratumoral microbiota remains uncharacterized in ACC. To bridge this gap, this study, for the first time, delineates the characteristics of the intratumoral microbiota in ACC. By exploiting the intratumoral microbiome data characterized by Poore et al. [5], we found that the intratumoral microbiome is associated with prognosis, host genomic events, and immune status in ACC. Additionally, microbial signatures could improve prognosis prediction compared with stage alone. Our study holds promise for a better understanding of the biology and for developing novel treatment strategies in ACC.

## RESULTS

### ACC harbors intratumoral microbes

**Figure 1:**
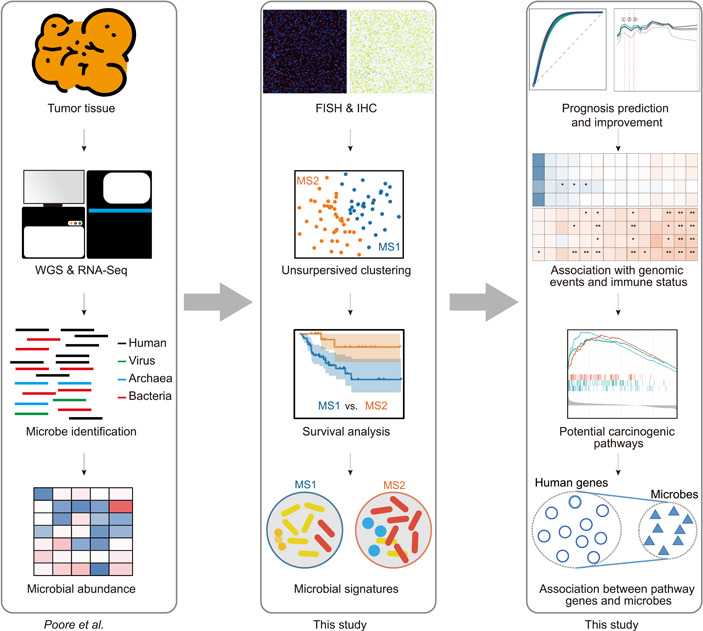
Overview of the analysis pipeline. The Cancer Genome Atlas primary tumors were subject to whole‐genome sequencing and RNA‐Seq, in which microbial abundance (comprising virus, archaea, and bacteria) was identified and normalized by Poore et al. (left panel). To confirm the presence of intratumoral bacteria, 37 adrenocortical carcinoma tissue microarray chips from the in‐house cohort were stained for fluorescence in situ hybridization and immunohistochemistry examination. Unsupervised clustering was performed on microbiome data to explore natural clusters of patients with distinct microbiomes, thereby influencing prognoses. The microbial signatures associated with prognosis were further determined (middle panel). Defined microbial signatures were tested to improve prognosis prediction. The intratumoral microbiome was found to be associated with immune status, genetic events, and molecular pathways (right panel).

To illuminate the intratumoral microbiota in ACC, we revisited and obtained the intratumoral microbial profiles in different cancers, which were processed by Poore et al. using whole‐genome sequencing (WGS) and RNA sequencing (RNA‐Seq) data from TCGA [5] (Figure 1). The intratumoral microbes comprised three types of microorganisms (i.e., viruses, archaea, and bacteria). Normalization and decontamination were also included according to different criteria, yielding five microbial abundance matrixes comprising no contaminants removed (NR), likely contaminants removed (LR), contaminants removed by sequencing “plate–center” combinations (CR), all putative contaminants removed (PR), and contaminants removed with most stringent filtering (SR).

**Figure 2:**
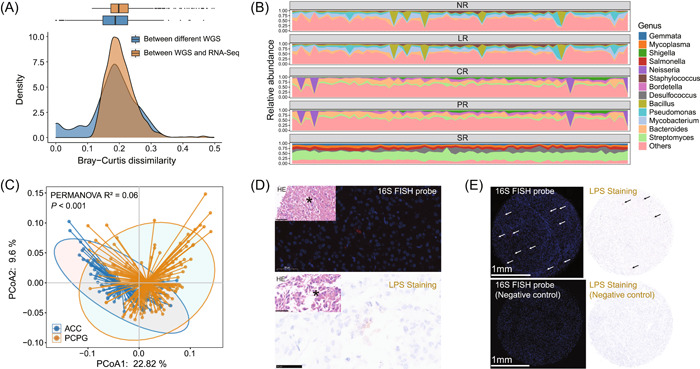
Adrenocortical carcinoma (ACC) harbors intratumor microbes. (A) Comparison of two different sequencing strategies in intratumor microbe detection for NR microbiome data. The Bray–Curtis dissimilarity was used to assess the differences in microbial communities identified from different WGS or from WGS and RNA‐Seq. The box plot on the top and the density plot on the bottom shows the Bray–Curtis dissimilarity distribution. (B) Principal coordinate analysis (PCoA) for ACC and PCPG, based on the Bray–Curtis dissimilarity. Permutational multivariate analysis of variance (PERMANOVA) gave the *p* value. (C) Relative abundance of microbes in each microbiome data with different levels of filtering. Only the top five genera of each microbiome data were shown. (D) Representative images of fluorescence in situ hybridization (FISH) staining of 16S rRNA (up) and immunohistochemistry (IHC) staining of LPS (down) in tissue containing 37 ACC samples. (E) Representative images of comparison between positive result (up) and the negative control (down) in FISH staining of 16S rRNA (left) and IHC staining of LPS (right). Arrows were used as indication for positive signals.

The ACC samples in TCGA consist of 79 RNA‐Seq data from the primary tumor of 79 patients, in which poly(A) enrichment of the mRNA was used when preparing RNA‐Seq libraries. This might have skewed the detection of intratumoral microbiota, as only partial prokaryotic mRNA was polyadenylated [16]. To explore this question, we first attempted to compare the difference in intratumoral microbiota identified by WGS and RNA‐Seq. In TCGA repository, 1837 patients contained both WGS and RNA‐Seq data, while 224 patients contained more than one WGS data set from the same primary tumor. We used Bray–Curtis dissimilarity to assess microbial communities within each individual, from WGS and RNA‐Seq or from different WGS data. Whereas we observed a slightly larger dissimilarity between WGS and RNA‐Seq than between different WGS data (Figure 2A, Supporting Information: Figure S1), no distinct double peak was found, implying that the bias from RNA‐Seq data was negligible for the downstream analysis.

We next examined the microbial profile in ACC, before and after decontamination. A total of 1794, 1552, 1406, 1284, and 169 genera were included in the NR, LR, PR, CR, and SR groups, respectively (Figure S2). While the genera *Mycobacterium* and *Pseudomonas* dominated NR and LR, *Bacteroides* and *Streptomyces* were the most abundant in PR and CR (Figure 2B). After the most stringent filtering, the genera *Streptomyces* and *Desulfococcus* were the most predominant in SR. As TCGA‐ACC cohort lacked paired normal control, we opted for adrenal pheochromocytoma (PCPG) as the control to exclude tumor context, in view of their similar site and surgical procedure. Principal coordinate analysis (PCoA) showed a significantly distinct microbiome composition between PCPG (*n* = 178) and ACC (*n* = 79) (permutational multivariate analysis of variance (PERMANOVA) test, *p* < 0.001), implying that the intratumoral microbiome was tumor type‐dependent (Figure 2C, Supporting Information: Figure S3).

To validate the presence of microbes in ACC, especially the most abundant bacteria, we stained the ACC tissue microarray chip (TMA) containing 37 samples from our in‐house cohort. With a universal probe against bacterial 16S rRNA, we adopted RNA fluorescence in situ hybridization (FISH) for detecting bacterial RNA in ACC tissue (Figure 2D). We also performed immunohistochemistry (IHC) staining against bacterial lipopolysaccharide (LPS), which is specific to detecting Gram‐negative bacteria (Figure 2D). Positive 16S rRNA and LPS staining was identified in 97.3% (36/37) and 83.8% (31/37) of ACC samples, respectively, indicating the actual presence of bacteria in ACC (Figure 2E).

### Intratumoral microbiome composition is associated with prognosis in ACC

**Figure 3:**
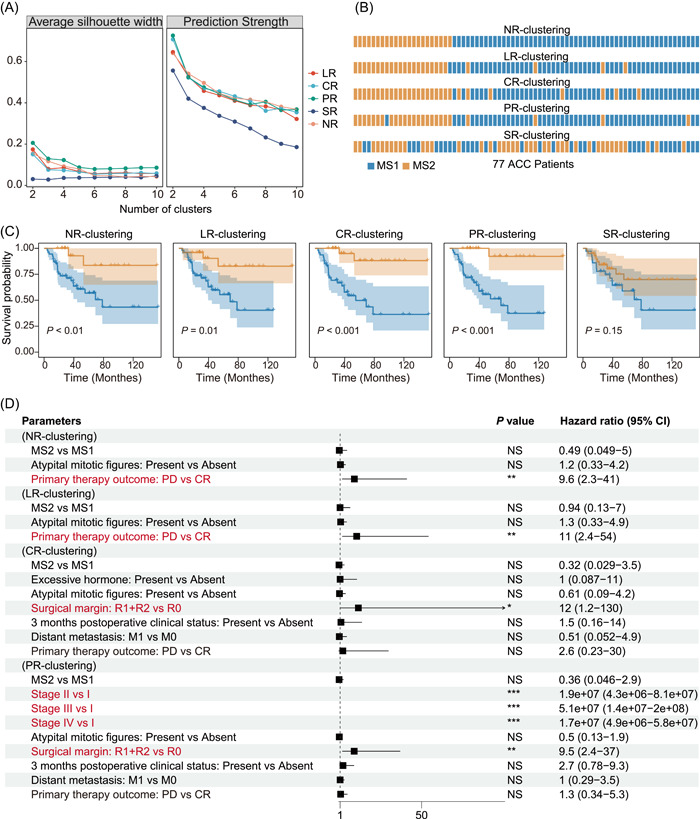
Intratumoral microbial composition in Adrenocortical carcinoma (ACC) is associated with prognosis. (A) Clustering scores of different cluster numbers. The quality of clustering was estimated by silhouette width and prediction strength, based on the CM distance. (B) Clustering results of 77 ACC patients for five microbiome data. Each cell represents one patient. (C) Kaplan–Meier plot of two clusters for five clusterings. The Log‐rank test gave the *p* value. (D) Multi‐variate cox analysis between confounders differentially distributed between MS1 and MS2 in different clustering situations. The clinical factors in red represent the significant ones independently influencing the prognosis. The significance was shown with asterisks. *p < 0.05; **p < 0.01; ***p < 0.001. NS, not significant.

Considering the possible relationship between the tumor microbiome and cancer prognosis [17], we sought to determine whether intratumoral microbiome composition could influence prognosis in ACC. We speculated that naturally distinct clusters of the intratumoral microbiome might be associated with ACC prognosis. For this purpose, an unsupervised clustering strategy, partition around medoids (PAM) clustering, was applied to five microbial abundance data of 77 patients (excluding two patients missing cancer stage) [18], according to Bray–Curtis (BC) dissimilarity, Jaccard distance, and a combined metric of two [19] (CM distance). Prediction strength (PS) and silhouette index (SI) were used to assess the cluster number and quality of the according clusters. We observed a low PS (<0.8) and SI (<0.5), signifying weak support for clustering (Figure 3A, Supporting Information: Figure S4), probably due to limited data size, potential contaminants, or no substantial difference in ACC intratumoral microbiome. However, three distances indicated a consensus of optimal cluster number, that is, two for all five abundance data sets, and the resulting clusters were identical regardless of the distances adopted (Supporting Information: Figure S5). This unsupervised clustering based on microbial composition, thus, grouped patients into two clusters. For the remainder of the paper, the two clusters of patients are termed MS1 (microbial signature 1) and MS2 cohorts. In the context of the five microbial abundance data sets, the two MS cohorts (MS1 vs. MS2) were 55 versus 22 (NR‐clustering), 52 versus 25 (LR‐clustering), 48 versus 29 (PR‐clustering), 52 versus 25 (CR‐clustering), and 36 versus 41 (SR‐clustering), respectively (Figure 3B). The NR‐clustering, LR‐clustering, PR‐clustering, and CR‐clustering data sets were relatively stable, whereas the SR‐clustering data set was entirely distinct from the other four clusters (Figure 3B, Supporting Information: Figure S5).

We then used principal coordinate analysis (PCoA), based on BC dissimilarity, to confirm the difference in the microbial community between each pair of MS1 and MS2. As expected, we found a significant difference (PERMANOVA test, *p* < 0.05) between the two MS cohorts in all four microbial abundance data sets when adopting NR‐clustering, LR‐clustering, PR‐clustering, or CR‐clustering, but not SR‐clustering (Supporting Information: Figure S6). The alpha diversity of the MS cohorts was also assessed using the Shannon index. Regardless of the four clusters (i.e., NR‐clustering, LR‐clustering, PR‐clustering, and CR‐clustering), MS1 displayed a significantly higher diversity (Wilcoxon signed‐rank test, *p* < 0.05) than MS2 for NR, LR, PR, and CR but not SR. In comparison, no significant difference was observed in any abundance data when adopting SR‐clustering (Supporting Information: Figure S7).

We then attempted to interrogate the prognosis difference between the two MS cohorts. To do this, we adopted the Kaplan–Meier estimate for examining the survival distribution [20], using the log‐rank test to test the difference between two clusters. A significantly higher survival outcome in MS2 than MS1 was observed when applying any of NR‐clustering (*p* < 0.01), LR‐clustering (*p* = 0.01), CR‐clustering (*p* < 0.001), and PR‐clustering (*p* < 0.001). In contrast, no survival difference was found using SR‐clustering (Figure 3C). These results suggest an association between intratumoral microbiome composition and prognosis in ACC.

Lastly, we assessed the clinical characteristics between the two MS cohorts. We found that the MS1 cohort (worsened prognosis) was enriched with atypical mitotic figures, persistent tumor‐bearing status, and primary therapy outcome in four clusters (NR‐clustering, LR‐clustering, PR‐clustering, and CR‐clustering) (Figure 3D, Supporting Information: Table S1). To determine whether the microbial composition could function as an independent prognostic biomarker, multivariate Cox analysis was performed, which showed that microbiota subtype, as well as atypical mitotic figures or 3 months postoperative clinical status, failed to predict prognosis independently in ACC cohorts. Of exploratory interest, individual microbiota that were potentially associated with some clinical variates (e.g., neoplasm status and primary therapy outcome) were analyzed using random forest analysis (Supporting Information: Table S2), in which some genera were shown to play important roles when taking clinical variables into consideration. For example, the genus *Lysinimicrobium* might relate to the primary therapy outcome, that is, complete response (CR) or progressive disease (PD).

### Intratumoral microbial signatures can improve prognosis prediction

**Figure 4:**
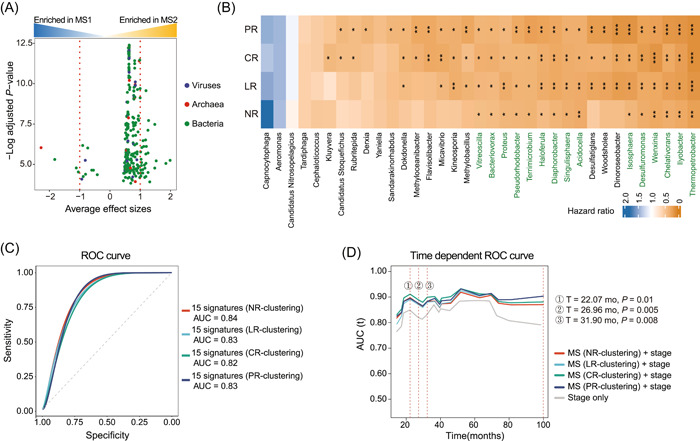
Intratumoral microbial signatures improve prognosis prediction. (A) Volcano plot of 309 consistently different genera between two clusters. *Y*‐axis is the log (FDR‐adjusted *p* values), and X‐axis is the average effect sizes. The effect size of each genus was the coefficient value in the linear model used in MaAsLin2. Each dot denotes one genus and is colored according to the superkingdom of the genus. Red dotted lines refer to the cutoff −1 or 1. (B) Heatmap showing hazard ratio of differently abundant 35 genus signatures. A hazard ratio of < 1 means a high abundance of the genus benefits prognosis. 15 genera highlighted in green were consistently significant. NR, microbiome data with no contaminants removed; LR, likely contaminants removed; CR, contaminants removed by sequencing “plate–center” combinations; PR, all putative contaminants removed; SR, contaminants removed with most stringent filtering. (C) ROC analysis of 15 genera‐based signature as predictive of overall status. The 15 differential genera identified were tested in aggregate. (D) Area under curve for overall survival between clinical stage alone and stage plus MS in a time‐dependent manner. The significance was shown with asterisks. *p <  0.05; **p < 0.01; ***p < 0.001.

Given that particular microbes can alter prognosis [21], we asked whether any microbes impinge on prognosis in ACC. As several clinical factors (e.g., age, gender, race, and clinical stage) might be associated with microbiome composition [22], we first sought to examine potential confounders of microbial signatures. The confounding effects of 14 clinical factors were quantified by the PERMANOVA test according to the BC dissimilarity. For NR and LR, we observed that the microbiome community composition varied with neoplasm status, 3 months postoperative clinical status, surgical margin, and atypical mitotic figures (false discovery rate (FDR)‐adjusted *p* < 0.05). In comparison, neoplasm status was the only confounding factor in CR and PR, and no clinical factor influenced the microbiome composition in SR (Supporting Information: Table S3). Even though other factors, including age, gender, and race, were not found to influence the microbiome community, we included them as strata for investigating the microbial signatures between the two clusters. We then used MaAsLin2 to determine the significantly different genera between two MS cohorts while adjusting for the six confounders [23]. Four clusters (i.e., NR‐clustering, LR‐clustering, CR‐clustering, and PR‐clustering) were applied to the five abundance data sets to ensure the robustness of potential microbial signatures. When applying any of the four clusters to SR data, no significantly different genus was found between two MS cohorts; in contrast, 309 genera were found to be consistently significantly different between two MS cohorts (FDR < 0.05) when applying any four clusters to any four abundance data sets (i.e., NR, LR, CR, or PR) (Figure 4A, Supporting Information: Figure S8 and Table S4).

To further determine the microbial signatures that might be associated with prognosis, we next stratified patients into two groups individually according to the median abundance of 35 genus features with an effect size <−1 or >1 (Figure 4A). The Cox proportional hazards regression model showed consistently significant hazard rates when stratifying patients individually according to 15 genera (Figure 4B). The difference in survival distribution was also confirmed by the Kaplan–Meier estimate (Supporting Information: Figures S9–S12), reinforcing that the high abundance of the 15 genera was closely associated with a favorable prognosis.

There are a limited number of reports on ACC prognostic biomarkers. Previous studies tried to construct a microarray‐based prognostic predictor and identified genes pair *BUB1B* and *PINK1* as optimal predictors (AUC = 0.83) of poor prognosis in ACC [24, 25]. To evaluate the predictive ability of overall status using these 15 genera, we performed area under curve (AUC) receiver operator characteristic (ROC) analysis. Surprisingly, the combination of 15 microbial taxa in our study observed an AUC of 0.84 in NR‐clustering, 0.83 in LR‐clustering, 0.82 in CR‐clustering, and 0.83 in PR‐clustering (Figure 4C). Furthermore, we found that incorporation of MS enhanced prognostic performance of stage alone at early months (*T* = 22.07 months, *p* < 0.05; *T* = 26.96 months, *p* < 0.05; *T* = 31.90 months, *p* < 0.05) of diagnosis (Figure 4D). This corresponds to another model that tumor microbial abundances, alone or in combination with tumor gene expression, can predict cancer prognosis and drug response to some extent [17].

### Intratumoral microbial composition is associated with host genomic events

**Figure 5:**
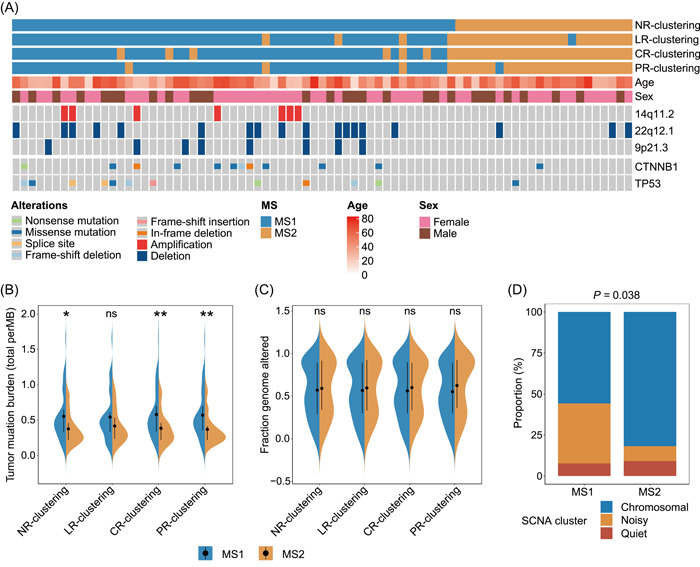
Intratumoral microbial composition is associated with host genomic events. (A) Waterfall plot showing the differentially distributed genomic events in adrenocortical carcinoma categorized by MS clustering. Violin plot showing the difference of (B) tumor mutation burden and (C) fraction genome altered between MS subtypes in different clustering situations. (D) Stacked bar plot showing *χ* ^2^ test of somatic copy number alteration cluster between MS1 and MS2 in NR clustering. The significance was shown with asterisks. *p <  0.05; **p < 0.01; ***p < 0.001; ns, not significant.

A large‐scale study identified five significantly mutated genes (SMGs) in ACC (*TP53*, *CTNNB1*, *MEN1*, *PRKAR1A*, and *RPL22*) and showed recurrent somatic copy number variations (CNVs) [4]. Therefore, we next attempted to depict the landscape of genetic mutations and CNVs of these patients. We compared the occurrence of various genomic events between two MS cohorts using the chi‐squared test (Supporting Information: Table S5), and observed a higher rate of *CTNNB1* and *TP53* mutations, as well as amplification of 14q11.2, loss of 22q12.1, and loss of 9p21.3 in MS1 with a worse prognosis (Figure 5A, Supporting Information: Figure S13). The pathway most affected by genomic alterations was the p53 signaling pathway (Supporting Information: Figure S14), which is the commonly activated pathway in most ACC tumors. We also compared tumor mutation burden (TMB) between two cohorts and found a higher TMB in the MS1 cohort, consistent with the previous finding that a high TMB level is associated with a worse prognosis [26] (Figure 5B). Although no difference in fraction genome alteration (FGA) was observed, MS1 was significantly enriched with a noisy somatic copy number alteration (SCNA) cluster, which is characterized by an aggressive disease phenotype [4] (Figure 5C,D, Supporting Information: Figure S15A).

### Intratumoral microbiota might play roles in an immunity‐dependent manner

**Figure 6:**
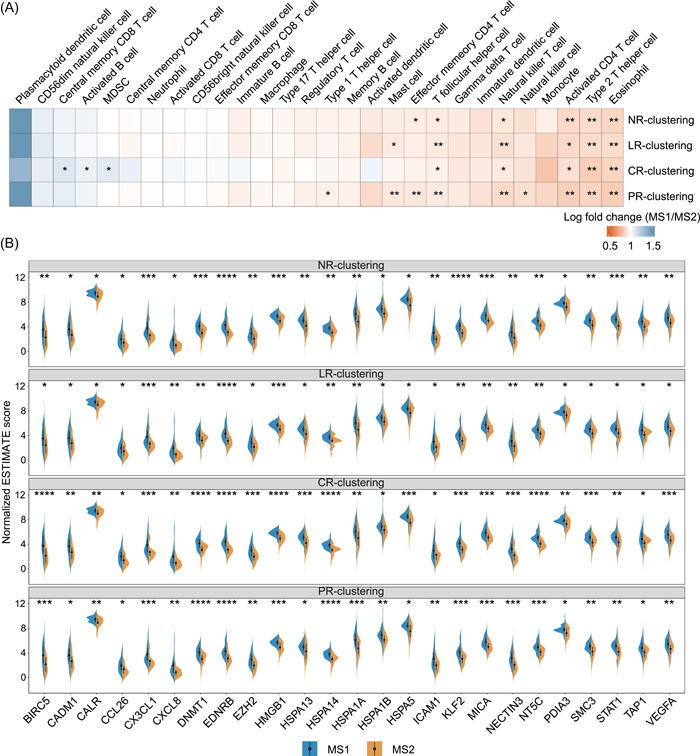
Intratumoral microbiota might play roles in an immunity‐dependent manner. (A) Heatmap showing the ratio of MS1/MS2 infiltrating estimations of 28 immune cells in tumor microenvironment. A ratio >1 means higher infiltration in MS1, while ratio <1 means lower in MS1. (B) Violin plot showing the difference of immune‐related gene expression between MS subtypes in different clustering situations. The significance was shown with asterisks. *p < 0.05; **p < 0.01; ***p < 0.001; ****p < 0.0001.

ACC is characterized by indolent immunity and immunotherapy resistance [27]. To evaluate the immune status within two MS cohorts, we scored the overall immune or stromal cells of the tumor microenvironment (TME) and revealed a lower immune estimation in MS1 when compared to MS2 (Supporting Information: Figure S16). More specifically, we estimated 28 immune cell types infiltrating and observed a lower proportion of tumor‐infiltrating lymphocytes (TILs) in the MS1 cohort (Figure 6A), including activated CD4^+^ T cells, natural killer T cells, type 2 T helper cells, and eosinophils. A difference in these immune cells was also observed in other solid tumors, such as breast and lung cancers [28, 29]. As many of intratumoral microbiota effects on the TME appear to suppress local antitumor immunity [30], we next interrogated the immune suppression genes related to multiple immune cells. Accordingly, we identified several genes, including CD8 T cell negatively related (*CX3CL1*) and T cell negatively related genes (*EZH2*, *DNMT1*, *EDNRB*, *ICAM1*, and *VEGFA*), which were upregulated in the MS1 cohort (Figure 6B).

### Intratumoral microbiota might activate carcinogenic pathways

**Figure 7:**
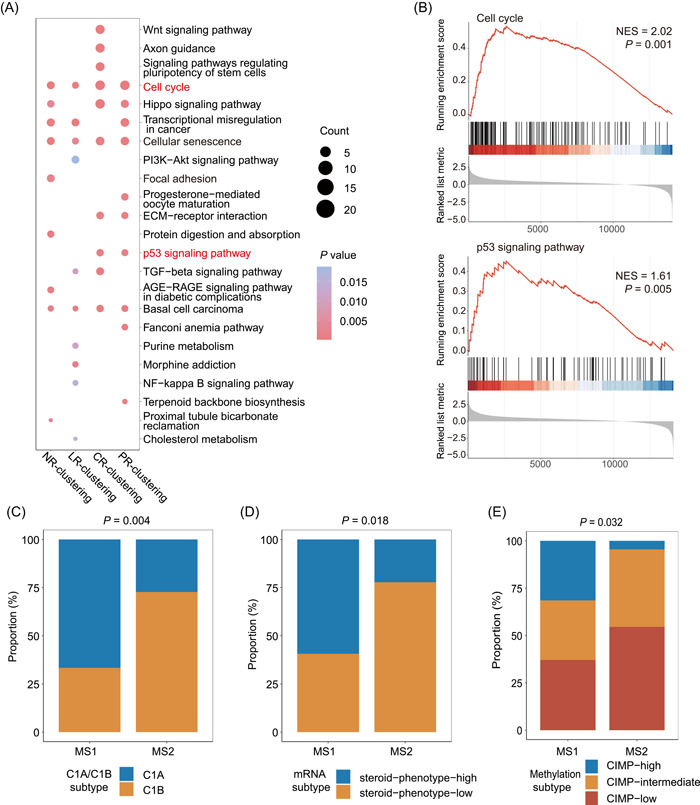
Intratumoral microbiota might activate carcinogenic pathways. (A) Dot plot showing the top 10 pathways enriched in MS1 using KEGG enrichment analysis in different clustering situations. The size of the dot represents the gene counts contributing to the pathway. (B) Enrichment plots showing increased expression of cell cycle and p53 signaling pathway gene sets in MS1 cohort. Stacked bar plot showing *χ* ^2^ test of (C) C1A/C1B cluster, (D) mRNA cluster, (E) methylation cluster proportions between MS1 and MS2 in NR clustering.

An insightful understanding of microorganism‐associated molecular pathways (MAMPs) is a standard procedure to identify the “friend or foe” role of the intratumoral microbiota. We first explored host transcriptomics in ACC to determine differentially expressed genes (DEGs) between two MS cohorts. KEGG pathway enrichment analysis of upregulated DEGs in MS1 (worse prognosis cohort) revealed the top 10 enriched pathways when applying four clusters (Figure 7A), with several interesting pathways overlapping. Considering the signaling pathways related to ACC progression and those related to microbiota reported elsewhere, the cell cycle and p53 signaling pathway attracted our attention. The upregulation of these two pathways in MS1 was also validated using GSEA (Supporting Information: Table S6), with NES being 2.02 (*p* = 0.001) and 1.61 (*p* = 0.005) for the cell cycle and p53 signaling pathway, respectively (Figure 7B). In another aspect, the MS1 cohort was enriched with an aggressive transcriptomic subtype of C1A and steroid phenotype high [4] (Figure 7C,D, Supporting Information: Figure S15B). The transcriptomic subtype of C1A was defined as malignant tumors showing a worse prognosis according to gene expression analysis in a study, which could be used as an independent prognostic biomarker in addition to pathology and tumor staging; this has been accepted as a robust predictor [31]. Additionally, MS1 showed a higher proportion of high DNA methylation level (Figure 7E, Supporting Information: Figure S15D), indicating a more malignant phenotype in MS1. However, we did not observe any association between the MS signatures and signatures of histology, miRNA, protein, or adrenocortical differentiation score (ADS) (Supporting Information: Figure S17A–D).

## DISCUSSION

In this study, we took advantage of microbial abundance generated by Poore et al. [5] to undertake a systematic investigation of the intratumoral microbiome in ACC. Using bacterial 16S rRNA FISH and LPS staining, we also validated the presence of ITB in our in‐house cohort. The unsupervised clustering method inferred two naturally distinct clusters of intratumoral microbiota in ACC, which were associated with overall survival. Incorporating microbial signatures could improve the predictive ability of disease status compared with that only based on the clinical stage. Additionally, the intratumoral microbiome was also found to be associated with host genomic events and immune status. In summary, our study provides deep insight into the intratumoral microbiome for ACC, and it could stimulate future studies on how the intratumoral microbiome could guide targeted therapies and immunotherapies.

The intratumoral microbiota has been discovered in most, if not all, human cancers, including in adjacent normal and deep tumor tissues, which are usually considered sterile. However, the composition of intratumoral microbiota varies drastically per different cancer types [5, 7, 32]. Consistent with previous studies of different solid tumors, we, for the first time, observed the presence of ITB in over 80–90% samples of ACC using 16S rRNA FISH and LPS staining. Whether residing within or close to the tumor and/or TILs, the intratumoral microbiota shows strong cancer type‐dependent characteristics [7, 32]. Indeed, we found a significant difference in intratumoral microbiomes between ACC and PCPG, although they are remarkably similar concerning the site and surgical procedure. Therefore, intratumoral microbiome uniqueness might serve as a biomarker for identification or diagnosis, as claimed by some scientists (Poore et al., etc.) [5, 7]. Additionally, emerging studies highlighted the intra‐tumoral microbial influence on more clinical phenotypes such as tumor relapse [33], tumor metastasis [6], and prognosis [21] of patients. Recently, the mechanism of the tumor‐resident microbiota affecting tumor biology has become a study hotspot. For example, tumor‐resident *F. nucleatum* triggers the GalNAc/autophagy/TBC1D5 signaling in oral squamous cell carcinoma (OSCC), driving tumor‐associated macrophage (TAM) formation, and OSCC progression [34]. Following this trend, our study proposes the prognostic role of the intratumoral microbiome, with the aim of comprehensively characterizing the potential biological roles, which can pave the way for a deeper study.

Unsupervised clustering is a potent machine learning technique to detect the naturally distinct groups in data sets, which has been widely used in various scientific studies, such as identifying cell types from single‐cell RNA sequencing data [35] and detecting enterotypes of the human gut microbiome [36]. Of note, several factors, such as clustering methodology and distance metrics, influence cluster detection [37], while no clustering method can perform optimally across all data sets. In this study, we applied PAM clustering to ACC microbiome data, optimally detecting two distinct clusters by measuring prediction strength and silhouette index. However, we observed a maximum prediction strength of less than 0.8, an empirical threshold of moderate support for clustering [38]. This may have been due to the small data size, potential contaminants, or no considerable difference in ACC intratumoral microbiome. In particular, potential universal contamination retained in the data set might decrease the power of cluster detection, as it would make the microbiome more similar. Despite this, we still observed a prognostic difference between two clusters, indicating the association between microbial community and prognosis.

Whether intratumoral microbiome plays a role in prognostics is quite a vital question in cancer biology. Previous pan‐cancer survival analysis based on the microbial signature revealed that ACC was one of the few cancer types where the intratumoral microbiota conferred an additional benefit to established staging systems for prognosis prediction [17]. In a landmark study by Qiao et al. [33], nasopharyngeal carcinoma (NPC) was shown to harbor intratumoral bacteria, whose composition was associated with NPC relapse, while the ITB load was associated with prognosis. Surprisingly, the microbiota‐based clustering in our study substantially differentiated the patients into subgroups with distinct clinical statuses, including tumor‐bearing status and immediate relapse of disease. We also proposed that 15 genera‐based signatures could serve as a prognostic marker, reaching over 0.8 of the AUC value in predicting prognosis. Some of the signatures have been revealed to be associated with solid tumors or other. For example, *Proteus mirabilis* localized preferentially in tumor tissues and remarkably suppressed primary tumor growth and pulmonary metastasis in breast tumor models. Genera *Isosphaera* and *Singulisphaera* belong to the phylum Planctomycetes, which has generally been reported to produce anticancer compounds [39, 40, 41]. Furthermore, compared to current tools, such as tumor stage in ACC, microbial clustering holds promise to enhance the performance of prognostic prediction, suggesting potential clinical transformation value.

DNA mutations are among the effects of the intratumoral microbiota on cancer development [42]. The microbiota has been reported to be a vital cause of DNA damage in several types of cancers, including gastrointestinal cancer [43, 44]. DNA damage caused by the microbiota further increases host genetic mutations, which may finally cause tumorigenesis [45]. In agreement with these findings, we found that, in addition to some copy number variations, some driver mutations such as *CTNNB1* and mutations have mostly occurred in the MS1 cohort with a worse prognosis. The *TP53* mutation is known for its correlations with the immunosuppressive microenvironment [46]. These genomic events were enriched in p53 signaling and the WNT pathway, which were also mentioned in other studies focusing on bacterial effectors influencing host cell signaling cascades [44, 47]. These consistent findings lend further credence to the causative role of the intratumoral microbiota in tumorigenesis. However, future studies are still warranted.

The activation of oncogenic pathways is another effect of the intratumoral microbiota. Many studies have uncovered that certain microbes could not only influence cytokines such as IL‐6 and TNF‐a directly or indirectly, but also activate the NF‐κB pathway or STAT3 pathway to promote tumor progression [45]. The association between specific taxa and the cell‐cycle pathway has previously been observed [48], and the genes or microRNAs in the cell cycle were found to be differentially expressed in conventional mice compared to those of germ‐free mice [49]. Moreover, the cell cycle is often associated with DNA damage. Our study found that the cell cycle was significantly enriched in the MS1 cohort, together with the p53 signaling pathway, which is one of the most activated pathways in ACC [4]. The p53 pathway was considered carcinogenic only in the presence of microbially‐produced gallic acid, suggesting a microbiome–functional genomic interaction [47]. We also explored the microbes significantly correlated with the genes belonging to these two pathways (Supporting Information: Figure S18), but we did not overstate the legitimacy of those findings. Given the limited sample size, overfitting could have substantially skewed the results.

ACC features indolent immunity and immunotherapy resistance [27], which refers to the suppressive immune status, showing either decreased amounts of immune cells or exhaustion of T cells, B cells, and NK cells with the downregulated tumor‐killing effect in the tumor microenvironment. Scientists have shown that dysbiosis in bacterial communities in the tumor environment can either cause a chronic, pro‐inflammatory immune response or modulate local immune surveillance by suppressing the antitumoral immune response [45, 50, 51]. Our study showed a lower proportion of overall immune or stromal infiltration, as well as TILs such as activated CD4^+^ T cells and natural killer T cells in MS1, indicating the TME suppression status. Further analysis highlighting the upregulation of a large number of immunosuppressive genes supported the above conclusion. This corresponds to the fact that MS1, enriched with C1A (characterized as a highly steroidogenic phenotype with mainly immune suppressor cortisol) [4], was shown to be associated with a worse prognosis. Mahata et al. [52] showed that tumors induce de novo steroidogenesis in T lymphocytes to evade antitumor immunity, confirming the immune suppression role of steroids. Furthermore, we discovered that excessive hormones are distinct between the two MS cohorts, suggesting the key role of steroidogenesis in the effects of the intratumoral microbiota on the tumor microenvironment.

Technically, unlike gut or body fluid microbial studies, shotgun metagenomics sequencing is not applicable for ITB detection given the extremely low biomass of intratumoral microbes [5, 7, 32]. However, several studies using the transcriptome 16S rRNA sequencing and FISH techniques have provided convincing evidence of the existence and location of the ITB. Fu et al. showed that cultured ITB from transgenic mice with spontaneous breast cancer could promote tumor metastasis via, surprisingly, mechanical reshaping by altering the cytoskeleton [6]. This culturomics approach could, however, hardly be extrapolated to ACC for us, as transgenic ACC mice are currently lacking, and organoids of ACC have rarely been reported, without even considering the dismal chance of capturing an ITB sequence from a patient's tumor samples given the astronomical host genetic materials. Wang et al. applied a metabolomics approach by evaluating the bacterial metabolite trimethylamine N‐oxide (TMAO) in breast cancer and traced the ITB that secreted TMAO [53]. Such an approach appears more feasible in ACC, with the only problem being the rarity of the disease.

The limitations of our study include the lack of external validation and a widely accepted decontamination protocol both in silico and in IHC. First, the rarity of ACC renders testing of sequencing techniques such as metagenomics and 16S rRNA extremely hard in a reasonable sample size. Functional analyses based on 16S rRNA sequencing and ex vivo culture for individual ITB are currently in progress to establish causal relationships between ITB and the host, which is still at the stage of tissue collection. Considering the rarity of the disease, it is hard to accomplish substantial specimen collection in a limited time. Additionally, the lack of fresh samples makes it hard to validate RNA‐Seq‐based signature designation and ITB isolation. Lastly, despite decontamination being considered, potential contaminants might still be retained across the entire analysis. How we processed our FFPE blocks warrants optimization. Nevertheless, our findings enable a better understanding of the biology of ACC.

## CONCLUSION

In summary, our study validated the presence of intratumoral microbes in adrenocortical carcinoma and characterized distinct microbiome composition that was associated with the prognosis, with 15 genera performing well in predicting prognosis. Intratumoral microbiota can distinguish the immune status of tumor microenvironment and can crosstalk with carcinogenic pathways such as p53 signaling and cell cycle signaling. Functional analyses warrant further exploration.

## METHODS

### Online data acquisition

Microbial abundance data and the corresponding metadata (file “Metadata‐TCGA‐Kraken‐17625‐Samples.csv”) were acquired from the online repository provided by Poore et al. [5] (ftp://ftp.microbio.me/pub/cancer_microbiome_analysis). Normalized and batch effect‐corrected microbiome data were directly adopted for bioinformatic analysis in this study. These microbiome data include data with raw counts (file “Kraken‐TCGA‐Raw‐Data‐17625‐Samples.csv”), data with Voom‐SNM normalization (file “Kraken‐TCGA‐Voom‐SNM‐Full‐Data.csv”), data with Voom‐SNM normalization and likely contaminants removed (file “Kraken‐TCGA‐Voom‐SNM‐Likely‐Contaminants‐Removed‐Data.csv”), data with Voom‐SNM normalization and putative contaminants removed (file “Kraken‐TCGA‐Voom‐SNM‐All‐Putative‐Contaminants‐Removed‐Data.csv”), data with Voom‐SNM normalization and contaminants removed by sequencing “plate–center” combinations (file “Kraken‐TCGA‐Voom‐SNM‐Plate‐Center‐Filtering‐Data.csv”), and data with Voom‐SNM normalization and most stringent filtering (file “Kraken‐TCGA‐Voom‐SNM‐Most‐Stringent‐Filtering‐Data.csv”). All microbiome data were composed of viruses, archaea, and bacteria, and they were measured at the genus level. The decontamination process was detailed in the original paper [5].

The gene expression profiles of RNA‐Seq data, including raw count and normalization to FPKM (fragments per kilobase of transcript per million fragments mapped), were downloaded from the UCSC Xena data set [54] (https://xenabrowser.net/datapages/). FPKM was further converted to TPM (transcripts per million). Genes absent in over 50% of the samples were filtered out. With a cutoff of 25% quantile of variance across samples, genes with low variance were also discarded, retaining 14,267 genes for downstream analysis. Gene sets for single sample gene set enrichment analysis (ssGSEA) were downloaded from the online data repository [55], and those for GSEA were acquired from the MSigDB database [56] (https://data.broadinstitute.org/gsea-msigdb/msigdb/release/7.5.1/).

Somatic mutation data in the form of mutation annotation format (MAF) were captured using the R package “TCGAbiolinks” (v2.16.4) [57]. Gistic2 copy number data were downloaded from the UCSC Xena data set [54] (https://xenabrowser.net/datapages/). Driver gene mutations or chromosomal spans with focal recurrent amplifications and deletions, together with molecular classification of different data types, were required from the online repository referenced by Zheng et al. [4]. Clinical data of TCGA‐ACC patients were downloaded from the cBioPortal data set [58] (https://www.cbioportal.org/).

### Unsupervised clustering for microbiome data

Unsupervised clustering was performed on five normalized ACC microbiome data using PAM clustering in R package “cluster” [18], a more robust clustering approach than *K*‐means clustering. This clustering algorithm relies on predefined distance metrics, which influence the detection of natural clusters in the microbiome [37]. The clustering variation can be reduced by a combined metric of the Bray–Curtis (BC) dissimilarity metric, which considers both microbial presence/absence and abundance, and unweighted UniFrac distance, which only considers the microbial presence/absence [19]. We, therefore, adopted three distances, namely, BC dissimilarity, Jaccard distance (only considering microbial presence/absence), and a combined metric of two with equal weight, termed CM distance, for PAM clustering. BC dissimilarity and Jaccard distance were computed by *vegdist* in R package “vegan” [59]; the CM distance was generated by the function *combMetric* in R package “MicobiomeCluster” (https://github.com/YushuShi/MicrobiomeCluster). We defined the combined metric (CM distance) as

$$d^{\mathrm{CM}}=0.5\;\times \;d^{\mathrm{BC}}\;+0.5\;\times \;d^{\mathrm{JD}},$$

where *d* ^CM^ is the CM distance, *d* ^BC^ is the Bray–Curtis dissimilarity matrix, and *d* ^JD^ is the Jaccard distance matrix. We assessed the optimal cluster number with the prediction strength (PS) [38] and silhouette index [60] (SI), where a score of ≥0.90 for PS or ≥0.75 for SI supports a strong clustering. PS was computed using the function *prediction.strength* in “fpc” package [38], and SI was computed using the *pam* function in “cluster” package. We also used the adjusted Rand index to compare the resulting clusters when applying distinct distance matrices to different microbiome data. In terms of the adjusted Rand index, a score of 0 refers to unrelated clusters and a score of 1 represents two identical clusters. This was performed using the function *adj.rand.index* in the R package “fossil” [61].

### Alpha diversity and beta diversity of microbial communities

The dissimilarity of microbial communities (beta diversity) between ACC and PCPG (pheochromocytoma and paraganglioma) or between different clusters was examined by principal coordinate analysis (PCoA) analysis based on BC dissimilarity. Permutational multivariate analysis of variance (PERMANOVA) based on BC dissimilarity with 999 permutations was used to compare the difference in microbial communities between groups, which was performed using *adonis2* in R package “vegan” [59]. Function *estimate_richness* in the package “phyloseq” was used to estimate the Shannon index (alpha diversity), representing the richness and evenness of microbial communities within each sample [62]. The statistical difference between groups was tested using Wilcoxon signed‐rank test, which was performed using the function *stat_compare_means* in “ggpubr” [63].

### Identification of microbial signatures associated with overall survival

PERMANOVA analysis with 999 permutations, based on BC dissimilarity, was performed to examine the effects of clinical factors on microbial communities. All *p* values were further adjusted for multiple comparisons with the FDR (false discovery rate) method [64]. The clinical factors with FDR‐adjusted *p* value < 0.05 were considered confounding factors. The differentially abundant microbes between two clusters were identified by MaAsLin2 (microbiome multivariable associations with linear models) while adjusting confounders, including age, gender, race, neoplasm status, 3 months postoperative clinical status, surgical margin, and atypical mitotic figures [23]. In the linear models, the cluster was considered a “fixed effect” and confounding factors were considered “random effects”. Only the genera with FDR‐adjusted *p* value < 0.05 were considered significantly different between two clusters.

### Survival analysis and prognostic prediction

The survival distributions between the groups were estimated with Kaplan–Meier curves, using the log‐rank test to test the difference between clusters. This was performed by *Surv* and *survfit* in package “survival” [65] and further plotted using *ggsurvplot* in “survminer” [66]. To explore the effects of microbial signatures on overall survival, we stratified patients into two groups according to the cutoff of the median abundance of each genus signature. Cox proportional hazard regression models were used for survival analysis between two groups of patients with low versus high abundance of a genus signature, which was finished by *coxph* in package “survival”. Hazard ratios (HR) and corresponding 95% confidence intervals (CIs) were calculated in the Cox models. The result was visualized by R package “forestploter” (v2.0.1) [67]. Receiver operating characteristic (ROC) curves were depicted using R package “pROC” (v1.18.0) [68] and “timeROC” (v0.4) [69], and the area under the curve (AUC) was used to assess predictive ability. Additionally, we used R package “randomForest” (v4.6‐14) [70] to identify microbial genera, most probably contributing to different clinical factors.

### Genomic analysis and visualization

Somatic variants were detected and analyzed by R package “maftools” (v2.4.12) [71]. Candidate genes were limited to driver events. To compare the frequency of variations between different groups, the *mafCompare* function was adopted. The waterfall plots showing the enriched pathways were visualized using the *oncoPrint* function in R package “ComplexHeatmap” (v2.13.1) [72].

### Transcriptomic analysis and visualization

The immune score and stromal score were assessed using R package “estimate” (v1.0.13) [73]. The ssGSEA was used to evaluate the relative proportion of 28 immune cells in tumor using R package “GSVA” (v1.36.3) [74], and the differences were examined by function *stat_compare_means* in package “ggpubr” (v0.4.0) [63]. Immune‐related genes were downloaded from the TIP web server (http://biocc.hrbmu.edu.cn/TIP/) [75]. Differentially expressed genes were detected using R package “limma” (v3.50.0) [76], with a cutoff of 1 for log‐transformed fold change. The Kyoto Encyclopedia of Genes and Genomes (KEGG) pathway enrichment of differential expression genes was performed using R package “clusterProfiler” (v3.16.1) [77]. Gene set enrichment analysis was performed using package “GSEABase” (v1.50.1) [78].

### Procrustes analysis

Procrustes analysis was performed using package “vegan” (v2.5‐7) [59] to evaluate the association between tumor microbiome composition and host gene expression. We calculated the BC dissimilarity based on the microbiome composition and gene expression matrix. Then, we used the nonmetric multidimensional scaling (NMDS) for dimension reduction and set *k* = 2 as the number of reduced dimensions or axes, whose result was the input for the rotations and statistical analysis in Procrustes analysis, with significance being tested with 9999 permutations using function *protest*.

### Sparse canonical correlation analysis (Sparse CCA) and enrichment analysis

For an integrative correlation analysis of two sets of measurements, we applied sparse CCA to identify group‐level correlations between paired host gene expression and microbiome data using the *CCA* function in R package “PMA” (v1.2.1) [79]. Hyperparameters were tuned with the *CCA.permute* function. The details of sparse CCA were described by Priya et al. [80]. We implemented the pathway enrichment analysis of host genes within each component and used Fisher's exact test for the significance test. The KEGG gene sets were downloaded from the MsigDB database using R package “msigdbr” (v7.5.1) [81]. The *p* values were corrected using the Benjamini–Hochberg method for controlling the false discovery rate (FDR), with FDR‐adjusted *p* < 0.1 considered significant.

### 16S rRNA staining (direct‐labeling RNA in situ hybridization)

To examine the presence of intratumoral bacteria in ACC, we performed 16S rRNA staining on an in‐house ACC tissue microarray (TMA) chip containing 37 formalin‐fixed samples. The tissues were resected adrenal tissues from patients with ACC receiving an adrenalectomy in Huashan Hospital affiliated with Fudan University. Then, they were formalin‐fixed and embedded in paraffin (wax) to create an FFPE block or paraffin block, which can be cut using a microtome to generate thin sections of tissue contained in paraffin to be stained. For downstream staining, the tissue sections were processed at 180°C for 4–6 h. Thorough sterilization of hood, blades, and relevant instruments was carried out. We adopted a direct labeling protocol because a strong background signal was observed using digoxin‐labeled probes. Briefly, paraffinized sections were dewaxed and dehydrated. Protease K was applied at room temperature. A working solution was then applied, and sections were incubated at 42°C for 2 h. After thorough rinsing with 0.2× SSC (saline–sodium citrate) buffer, 100 μM of EUB338‐cy5 probes (sequence: 5′–GCTGCCTCCCGTAGGAGT–3′) diluted in 1 μM of working solution were mounted and incubated at 42°C for 12–18 h. The procedure culminated with DAPI (1:500) staining. Scrambled probes were used as a negative control, and paraffin on the same tissue block was used as a contamination control.

### Immunohistochemistry (IHC) of lipopolysaccharide (LPS)

The same TMA block was used for IHC staining against bacterial lipopolysaccharide (LPS). Briefly, the block was sliced at 5 µm and mounted, followed by deparaffinization and hydration. After antigen restoration, the section was blocked with 3% hydrogen peroxide. Goat serum was applied, and the primary antibody against *Escherichia coli* LPS (Abcam, ab35654) was applied at a dilution of 1:200 overnight, followed by a mouse anti‐goat antibody (Abcam, ab205719). Diaminobenzidine (DAB) was applied, and slides were counterstained with hematoxylin. The procedure conformed to the ethics waiver regulation of our institute (Huashan Institutional Review Board, HIRB), as reported previously [82].

### Statistical analysis

All analyses and visualizations were performed via R software (v4.0.2) unless otherwise specified. We used Student's *t*‐test for parametric statistical testing and chi‐squared test or Fisher's exact test for nonparametric statistical testing between groups. We used NS (*p* > 0.05), *0.05 < *p* < 0.01, **0.01 < *p* < 0.001, and *** *p* <  0.001 to indicate the significance levels of *p* values in this paper.

## AUTHOR CONTRIBUTIONS

**Chenchen Feng**: Conceptualization; writing—review and editing. **Yong‐Xin Li**: Methodology; writing—review and editing. **Yuqing Li**: Data curation; data analysis; writing. **Dengwei Zhang**: Data analysis; writing—original draft. **Minghua Wang**: Visualization; investigation. **Haowen Jiang**: Supervision. All authors have read and approved the final manuscript.

## CONFLICT OF INTEREST STATEMENT

The authors declare no conflict interest.

## Supporting information

Supporting information.

Supporting information.

## Data Availability

All data from the current study was retrieved from public genomic data sets and no new data set was generated. All code used to produce this study is linked to the GitHub release (https://github.com/ZhangDengwei/ACC_Project). Supplementary materials (figures, tables, scripts, graphical abstract, slides, videos, Chinese translated version and update materials) may be found in the online DOI or iMeta Science http://www.imeta.science/
