# EXPRESSION OF CONCERN: Intratumoral Microbiota is Associated with Prognosis in Patients with Adrenocortical Carcinoma

**DOI:** 10.1002/imt2.255

**Published:** 2024-12-09

**EXPRESSION OF CONCERN**: Y. Li, D. Zhang, M. Wang, H. Jiang, C. Feng, and Y.–X. Li, “Intratumoral Microbiota is Associated with Prognosis in Patients with Adrenocortical Carcinoma,” *iMeta* 2, no. 2 (2023): e102, https://doi.org/10.1002/imt2.102.

This Expression of Concern is for the above article, published online on 05 April 2023 in Wiley Online Library (wileyonlinelibrary.com), and has been published by agreement between the journal Editors‐in‐Chief, Shuang‐Jiang Liu and Jingyuan Fu; iMeta Science; and John Wiley & Sons Australia, Ltd.

The above article utilized data partially derived from Poore et al. [1], which was later retracted due to data analysis errors [2]. In light of this retraction and the resulting uncertainty regarding the data, readers are advised to interpret the results of the present study with caution.

The journal team and the publisher are currently investigating the concerns to determine whether it affects the conclusions of the article. The authors have agreed to re‐analyze their data. In the meantime, the journal has decided to issue an Expression of Concern to inform and alert the readers.
