# Supplementary material for: Intratumoral microbiota is associated with prognosis in patients with adrenocortical carcinoma
Source: Imeta. 2023 Apr 5;2(2):e102. doi: 10.1002/imt2.102 (PMC10989844; doi:10.1002/imt2.102)
Supplement: Supplementary file 1 — Supporting information. [file IMT2-2-e102-s002.docx]

**Intratumoral microbiota is associated with prognosis in patients with adrenocortical carcinoma**

Running title: Intratumoral microbiota in ACC

Yu-qing Li^1#^, Deng-wei Zhang^2,3#^, Ming-hua Wang^1^, Hao-wen Jiang^1^*, Chen-chen Feng^1^*, and Yong-xin Li^2,3^*

^1^Department of Urology, Huashan Hospital, Fudan University, Shanghai 200040, China

^2^Department of Chemistry and The Swire Institute of Marine Science, The University of Hong Kong, Pokfulam Road, Hong Kong SAR, China.

^3^ Southern Marine Science and Engineering Guangdong Laboratory (Guangzhou), Guangzhou, China

^#^Equal contributors

*To whom correspondence may be addressed.

Email: [drfengchenchen@163.com](mailto:drfengchenchen@163.com) (Chen-chen Feng); [yxpli@hku.hk](mailto:yxpli@hku.hk) (Yong-xin Li)

[Figure S1. Comparison of two different sequencing strategies in intratumor microbe detection. 3](#_Toc124258018)

[Figure S2. The number of microbes detected before and after decontamination. 4](#_Toc124258019)

[Figure S3. Principal coordinate analysis (PCoA) of microbial compositions between ACC and PCPG. 5](#_Toc124258020)

[Figure S4. Clustering scores for different cluster numbers. 6](#_Toc124258021)

[Figure S5. Adjusted rand index between different clustering results. 7](#_Toc124258022)

[Figure S6. Difference of beta diversity between two clusters. 8](#_Toc124258023)

[Figure S7. Difference of alpha diversity between two clusters. 9](#_Toc124258024)

[Figure S8. Intersections among the significantly different microbes between two clusters in different microbiome data. 10](#_Toc124258025)

[Figure S9. Survival distribution based on the abundance levels of 15 microbial signatures in NR data. 12](#_Toc124258026)

[Figure S10. Survival distribution based on the abundance levels of 15 microbial signatures in LR data. 13](#_Toc124258027)

[Figure S11. Survival distribution based on the abundance levels of 15 microbial signatures in CR data. 14](#_Toc124258028)

[Figure S12. Survival distribution based on the abundance levels of 15 microbial signatures in PR data. 15](#_Toc124258029)

[Figure S13. Landscape of copy number variations (CNVs) and driver mutations between two clusters (MS1 & MS2) in ACC. . 16](#_Toc124258030)

[Figure S14. The p53 pathway-related genomic events were enriched in MS1. 17](#_Toc124258031)

[Figure S15. The correlation between MS and clinical parameters (significant in four clusterings). 18](#_Toc124258032)

[Figure S16. Immune estimation score between two clusters. 19](#_Toc124258033)

[Figure S17. The correlation between MS and clinical parameters (not significant in four clusterings). 20](#_Toc124258034)

[Figure S18. Links between gut microbial taxa and host genes in cell cycle pathway and p53 signaling pathway. 21](#_Toc124258035)


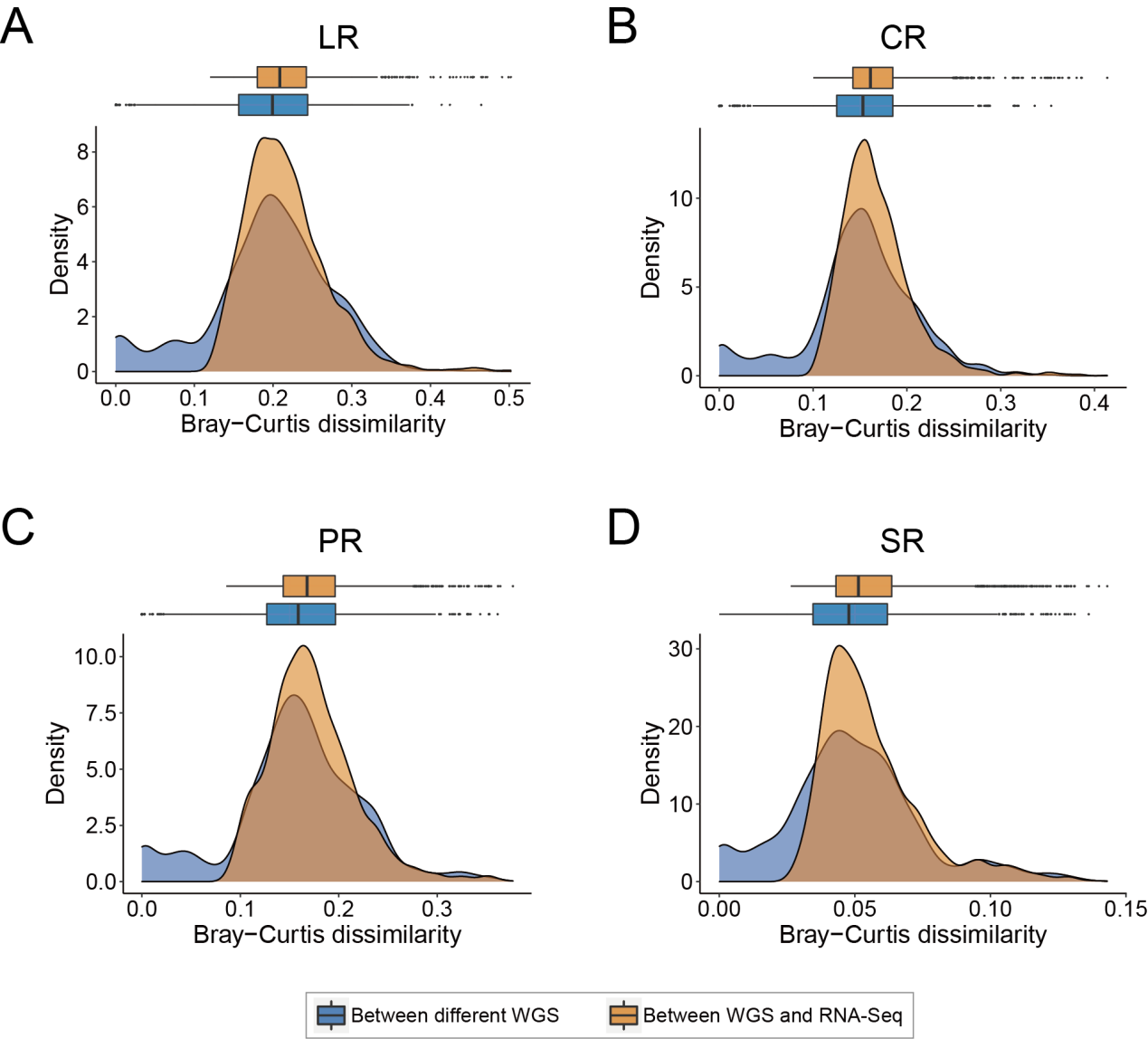


Figure S1. Comparison of two different sequencing strategies in intratumor microbe detection. For each patient, Bray-Curtis dissimilarity was used to assess the differences in microbial communities identified from different WGS or from WGS and RNA-Seq. The box plot on the top and the density plot on the bottom shows the distribution of Bray-Curtis dissimilarity assessed in two comparisons. No distinct double peaks were observed between the two comparisons, for microbiome data LR (**A**), CR (**B**), PR (**C**) and SR (**D**), implying the bias derived from RNA-Seq in microbe detection is limited. LR: Likely contaminants removed; CR: contaminants removed by sequencing “Plate-Center” combinations; PR: all putative contaminants removed; SR: contaminants removed with most stringent filtering.


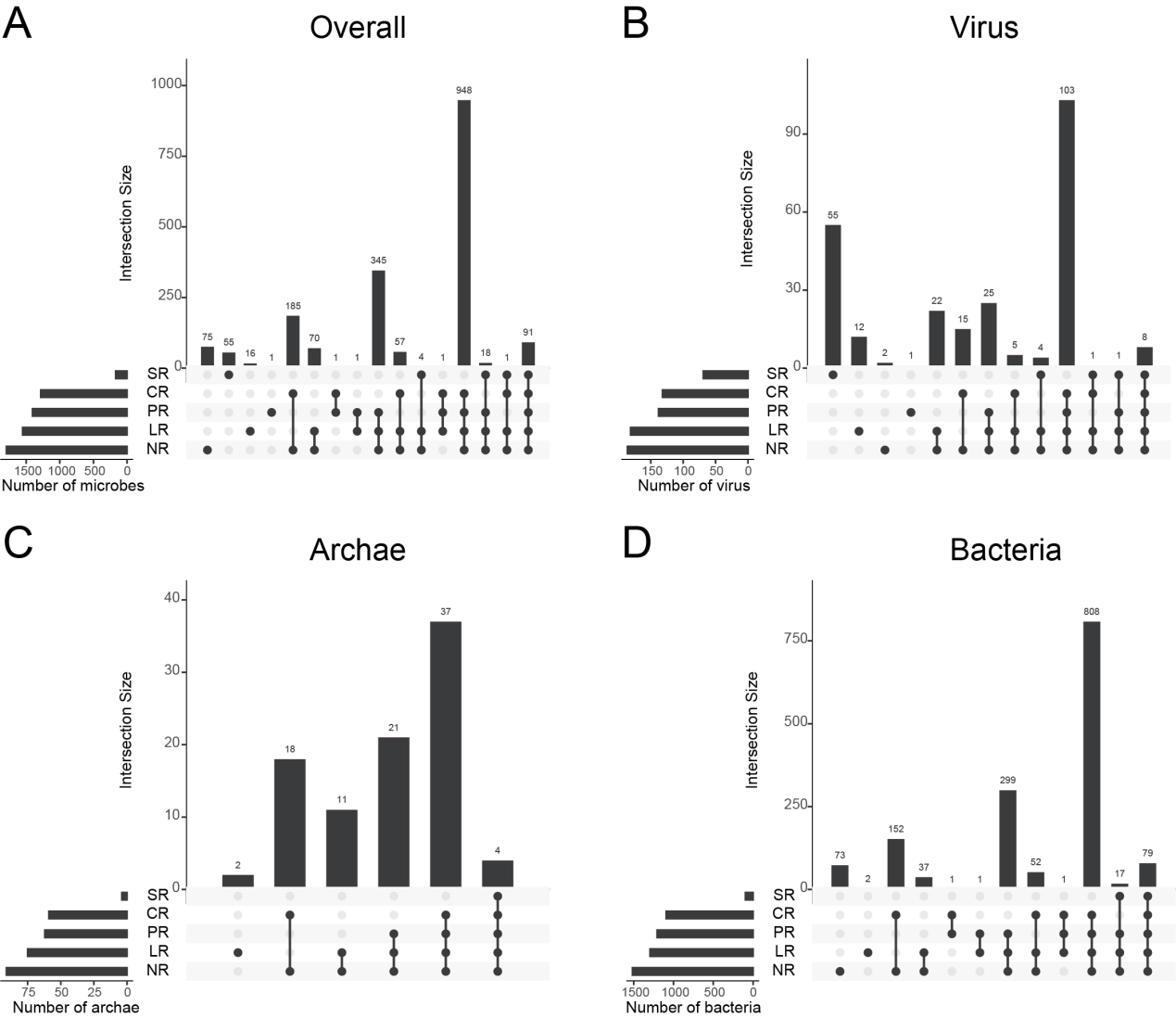


Figure S2. The number of microbes detected before and after decontamination. The bar plot on the left refers to the number of microbes (overall (**A**), virus (**B**), archaea (**C**), bacteria (**D**)) included in five microbiome data. The bar plot on top depicts the size of each intersection. The dark dots on the bottom denote which sets are included in each intersection and are connected by lines for better visibility. NR: no contaminants removed; LR: Likely contaminants removed; CR: contaminants removed by sequencing ‘Plate-Center’ combinations; PR: all putative contaminants removed; SR: contaminants removed with most stringent filtering.


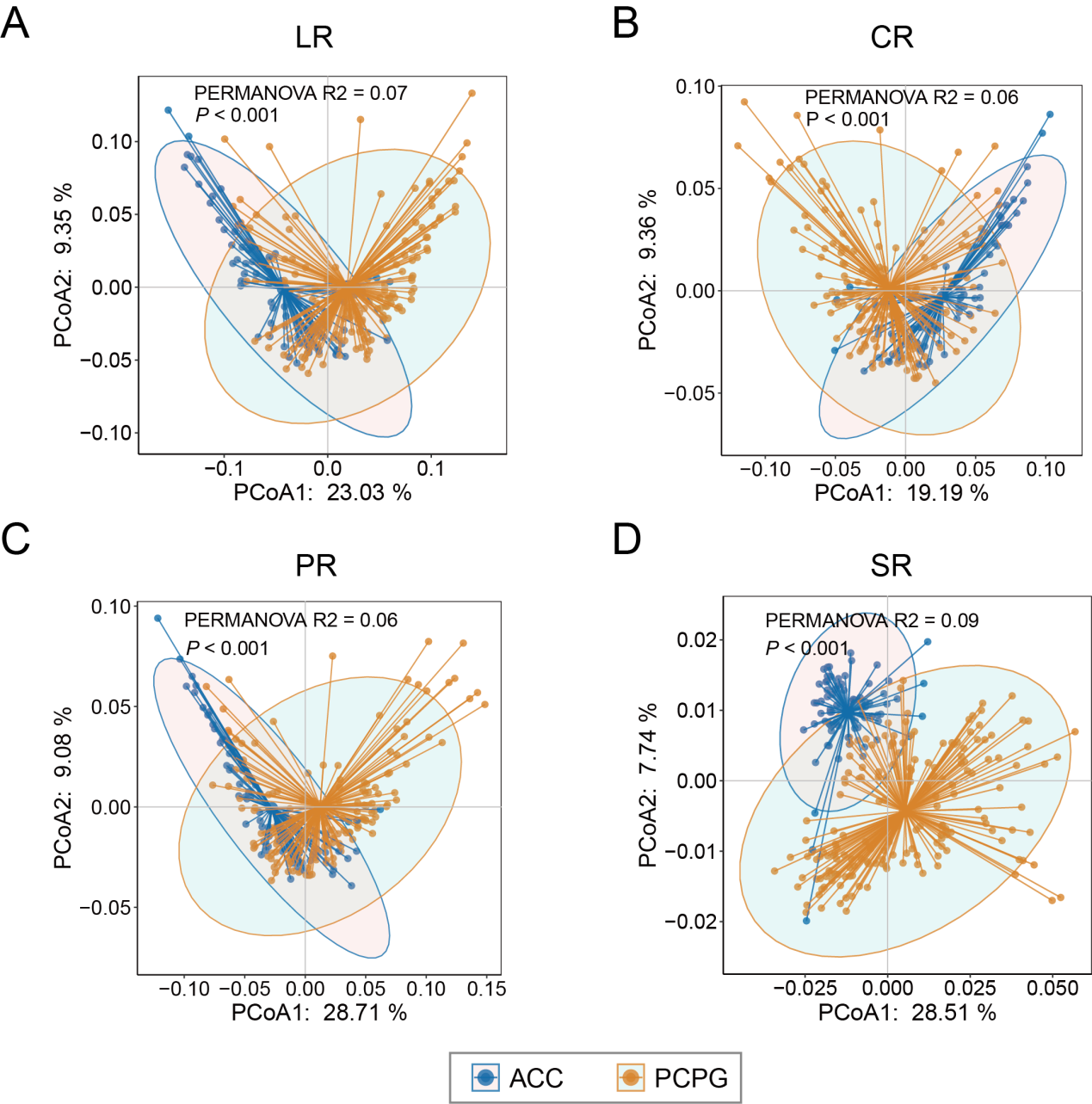


Figure S3. Principal coordinate analysis (PCoA) of microbial compositions between ACC and PCPG. The dissimilarity of microbial compositions was estimated by Bray-Curtis dissimilarity in five microbiome data, namely LR (**A**), CR (**B**), PR(**C**), and SR (**D**). *P* value was calculated by permutational multivariate analysis of variance (PERMANOVA). R2 denotes the variance of microbial difference explained by different cancer. LR: Likely contaminants removed; CR: contaminants removed by sequencing ‘Plate-Center’ combinations; PR: all putative contaminants removed; SR: contaminants removed with most stringent filtering.


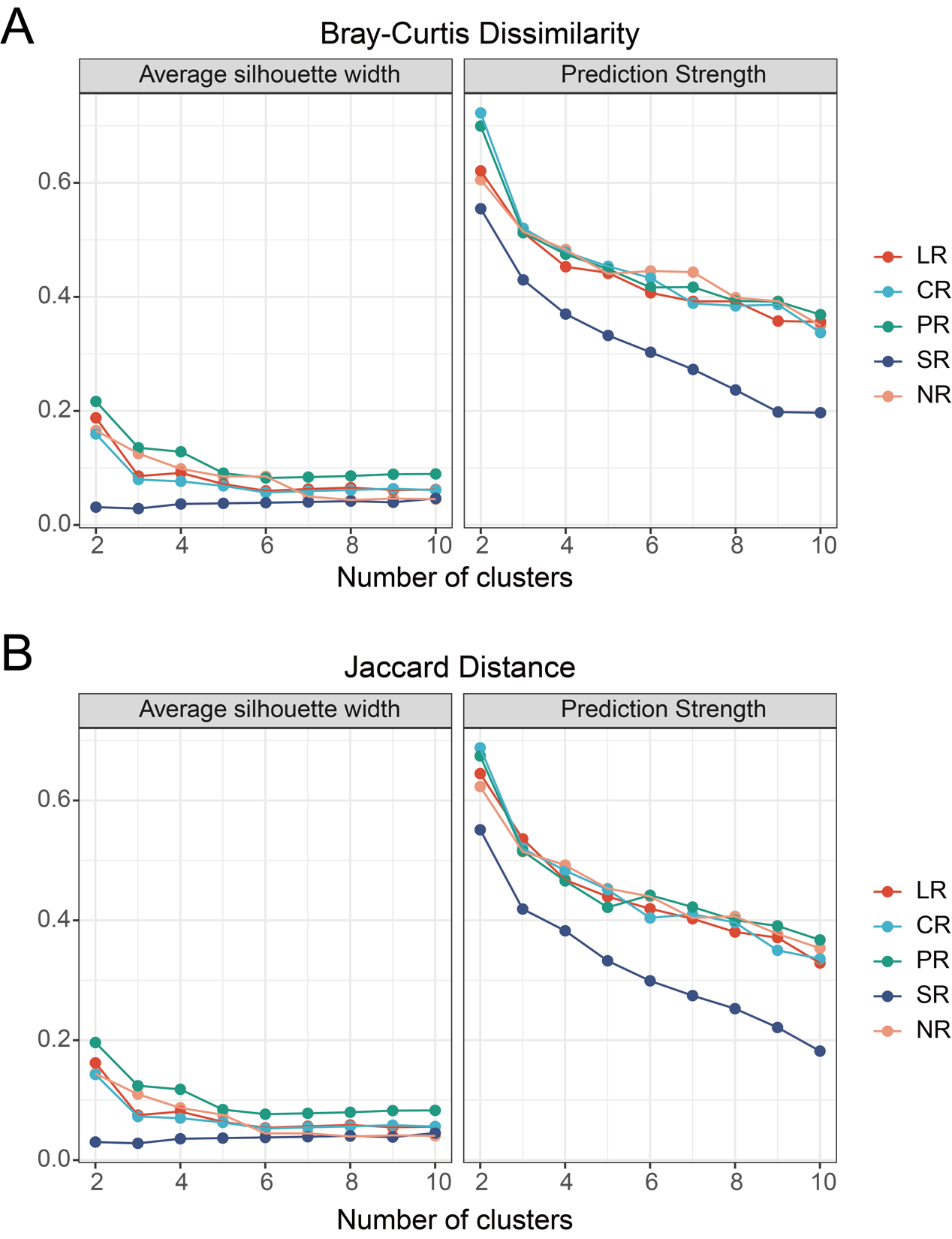


Figure S4. Clustering scores for different cluster numbers. The clustering quality was assessed using prediction silhouette index and strength, based on (**A**) Bray-Curtis dissimilarity and (**B**) Jaccard distance. The clustering number in five microbiome data (i.e., LR, CR, PR, SR, and NR) was respectively examined. NR: no contaminants removed; LR: Likely contaminants removed; CR: contaminants removed by sequencing ‘Plate-Center’ combinations; PR: all putative contaminants removed; SR: contaminants removed with most stringent filtering.


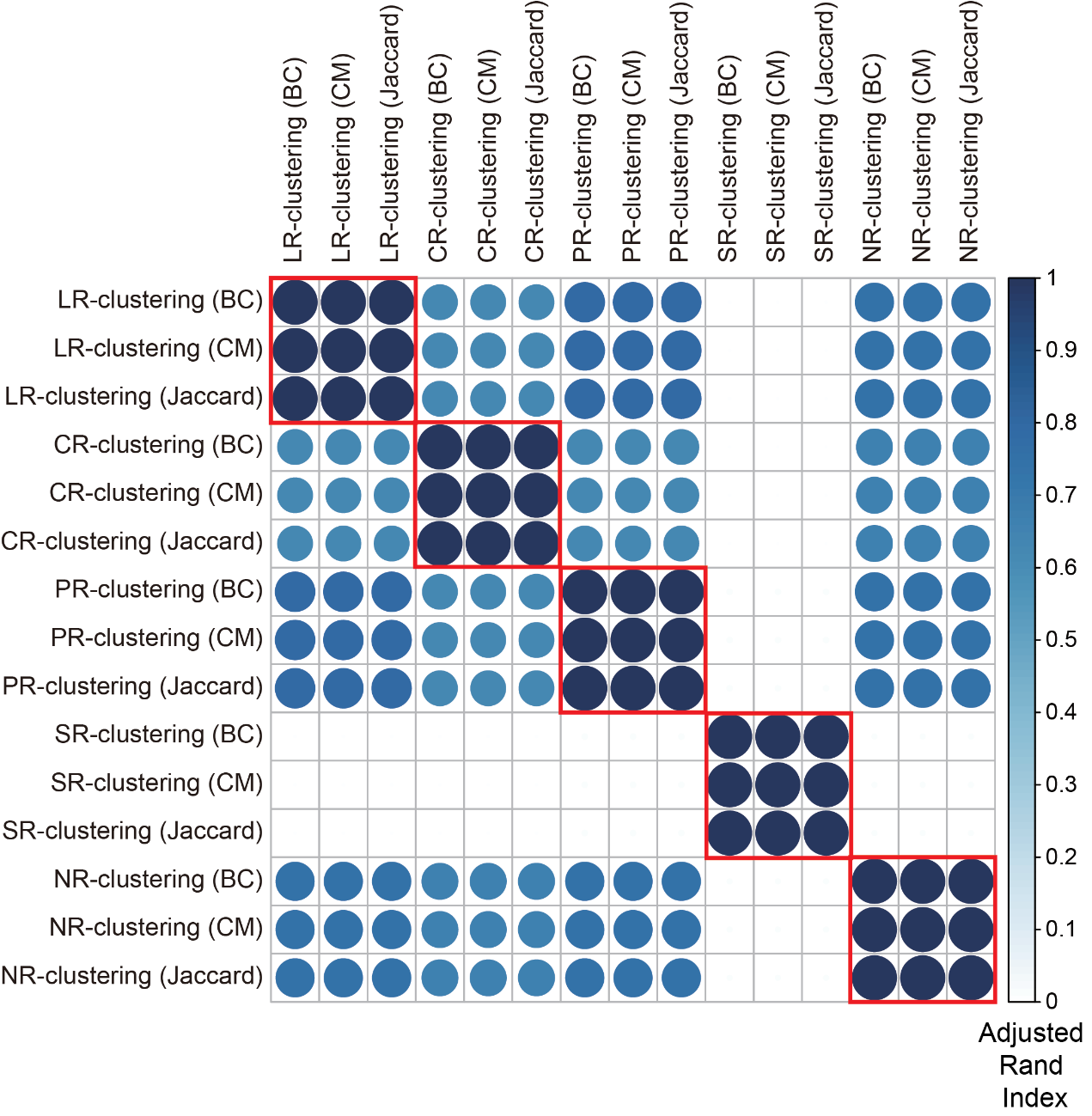


Figure S5. Adjusted rand index between different clustering results. Adjusted rand index was used to assess the similarity between two clustering results, where a score of 1 refers to identical clustering, and a score of 0 represents random and irrelevant clustering. Partition around medoids (PAM) clustering was applied to five microbiome data based on three distances: Bray–Curtis (BC) distance, Jaccard distance, and a combined metric of two (CM distance). The red rectangle indicates an identical clustering result with a score of 1 when clustering one microbiome data based on three distances.


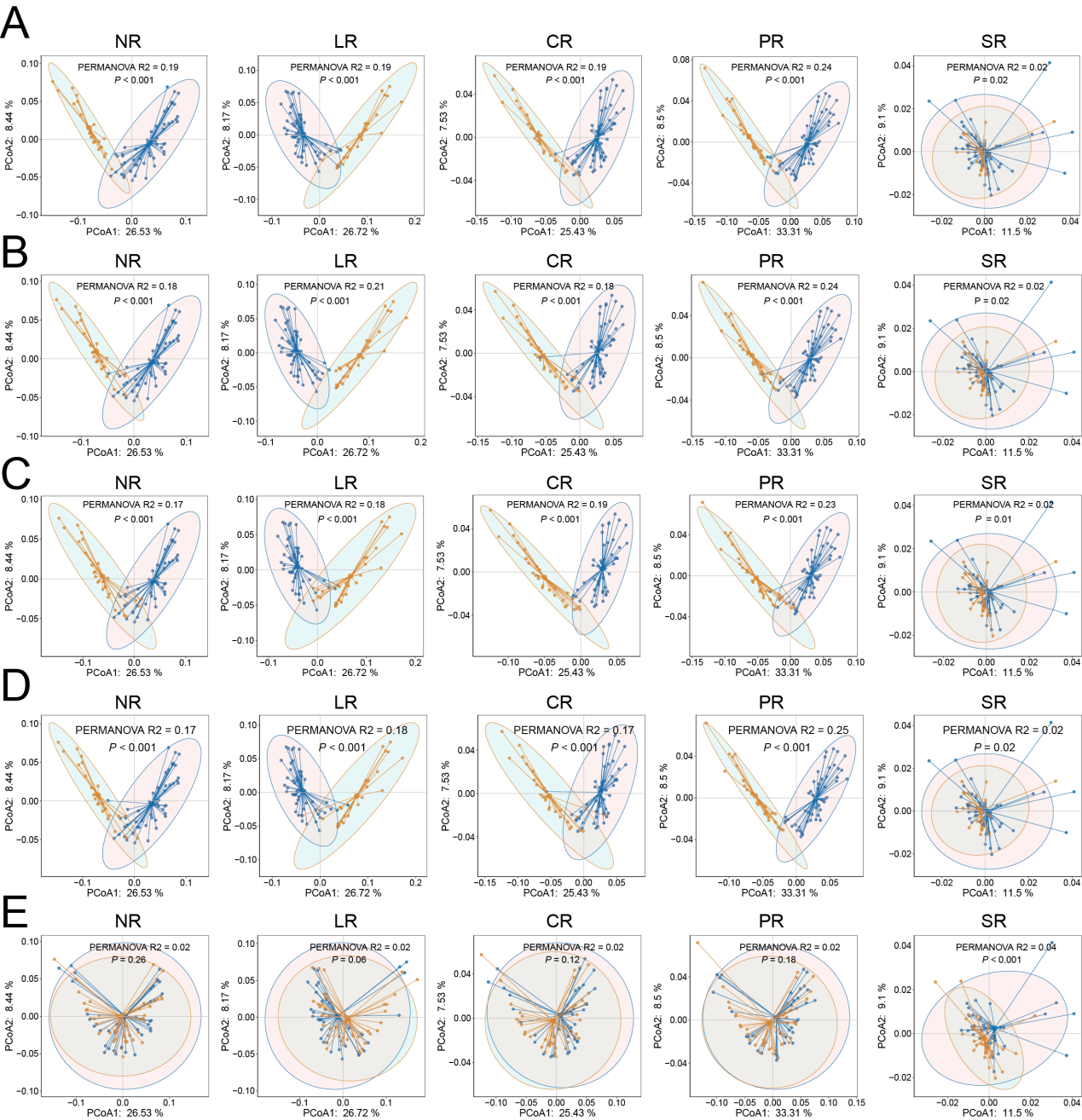


Figure S6. Difference of beta diversity between two clusters. Principal coordinate analysis (PCoA) was performed on five microbiome data when adopting NR-clustering (**A**), LR-clustering (**B**), CR-clustering (**C**), PR-clustering (**D**), and SR-clustering (**E**). It was based on the Bray-Curtis dissimilarity. *P* value was calculated by permutational multivariate analysis of variance (PERMANOVA) with 999 permutations. R2 denotes the variance of microbial difference explained by different clusters. NR: no contaminants removed; LR: Likely contaminants removed; CR: contaminants removed by sequencing ‘Plate-Center’ combinations; PR: all putative contaminants removed; SR: contaminants removed with most stringent filtering.


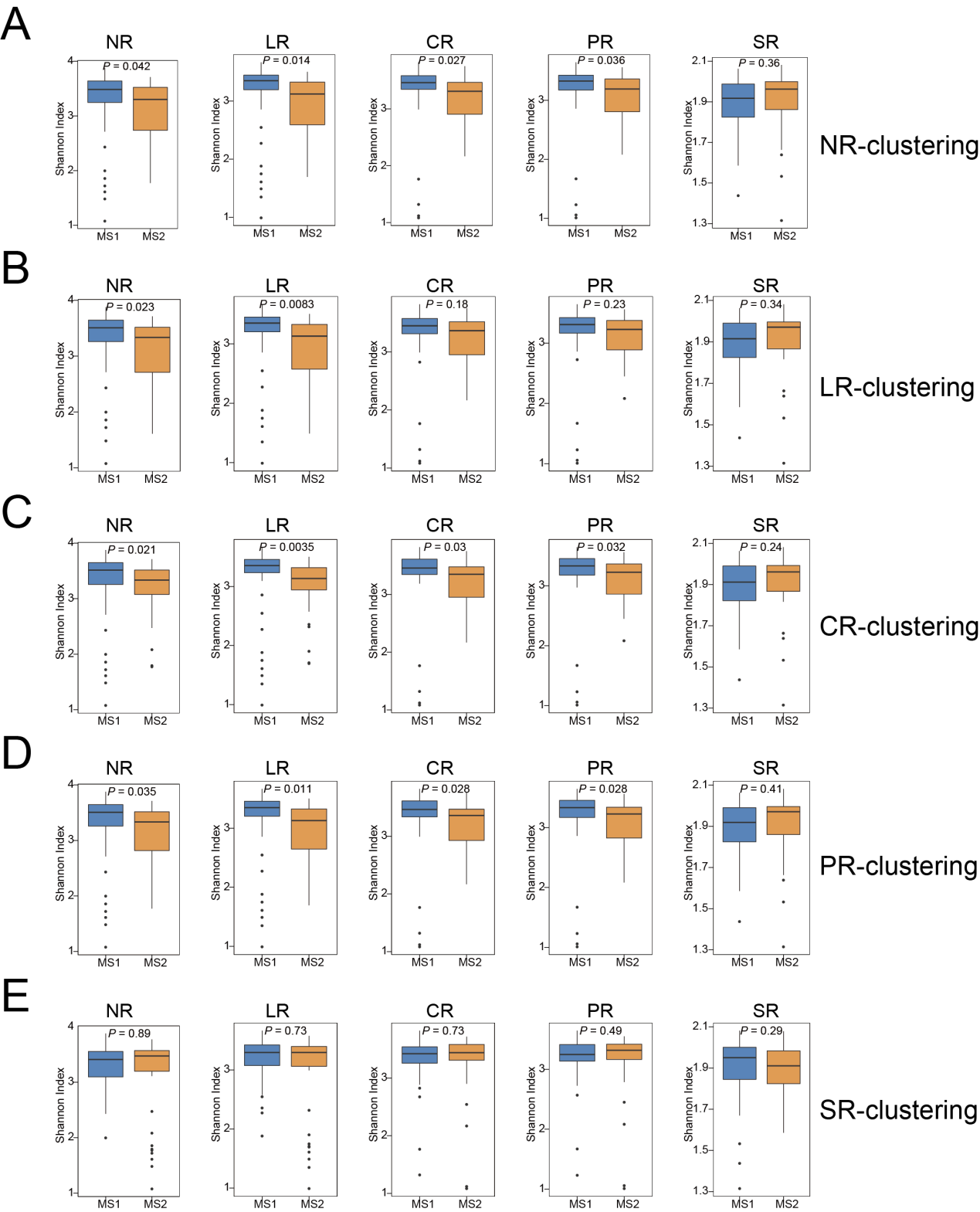


Figure S7. Difference of alpha diversity between two clusters. Alpha diversity estimated by the Shannon index in each cluster was examined when applying different clustering (i.e., NR-clustering (**A**), LR-clustering (**B**), CR-clustering (**C**), PR-clustering (**D**), and SR-clustering (**E**)) to five microbiome data. The comparison between the two clusters was performed using Wilcoxon rank-sum test. NR: no contaminants removed; LR: Likely contaminants removed; CR: contaminants removed by sequencing ‘Plate-Center’ combinations; PR: all putative contaminants removed; SR: contaminants removed with most stringent filtering.


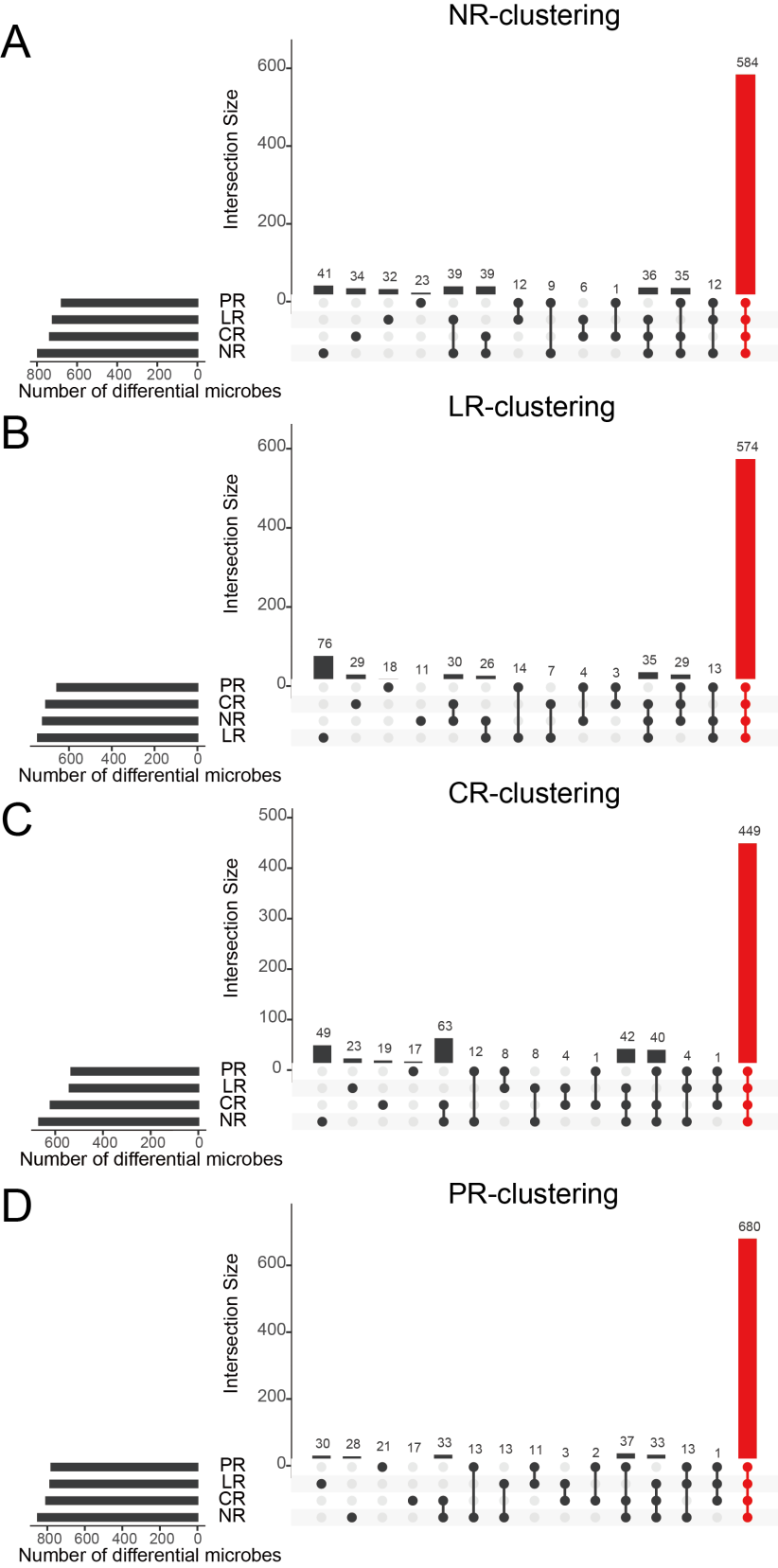


Figure S8. Intersections among the significantly different microbes between two clusters in different microbiome data. The differentially abundant genera were identified by MaAsLin2 (Microbiome Multivariable Associations with Linear Models). Only genera with FDR-adjusted *P* values < 0.05 were considered significantly different between the two clusters. Four clusterings, namely NR-clustering (A), LR-clustering (B), CR-clustering (C), and PR-clustering (D), were applied to four microbiome data respectively. The bar plot on the left depicts the number of differential genera identified in four microbiome data when adopting one clustering. The bar plot on top shows the size of each intersection. The dark dots on the bottom denote which sets are included in each intersection and are connected by lines for better visibility. The intersection of four microbiome data was highlighted in red. NR: no contaminants removed; LR: Likely contaminants removed; CR: contaminants removed by sequencing ‘Plate-Center’ combinations; PR: all putative contaminants removed; SR: contaminants removed with most stringent filtering.


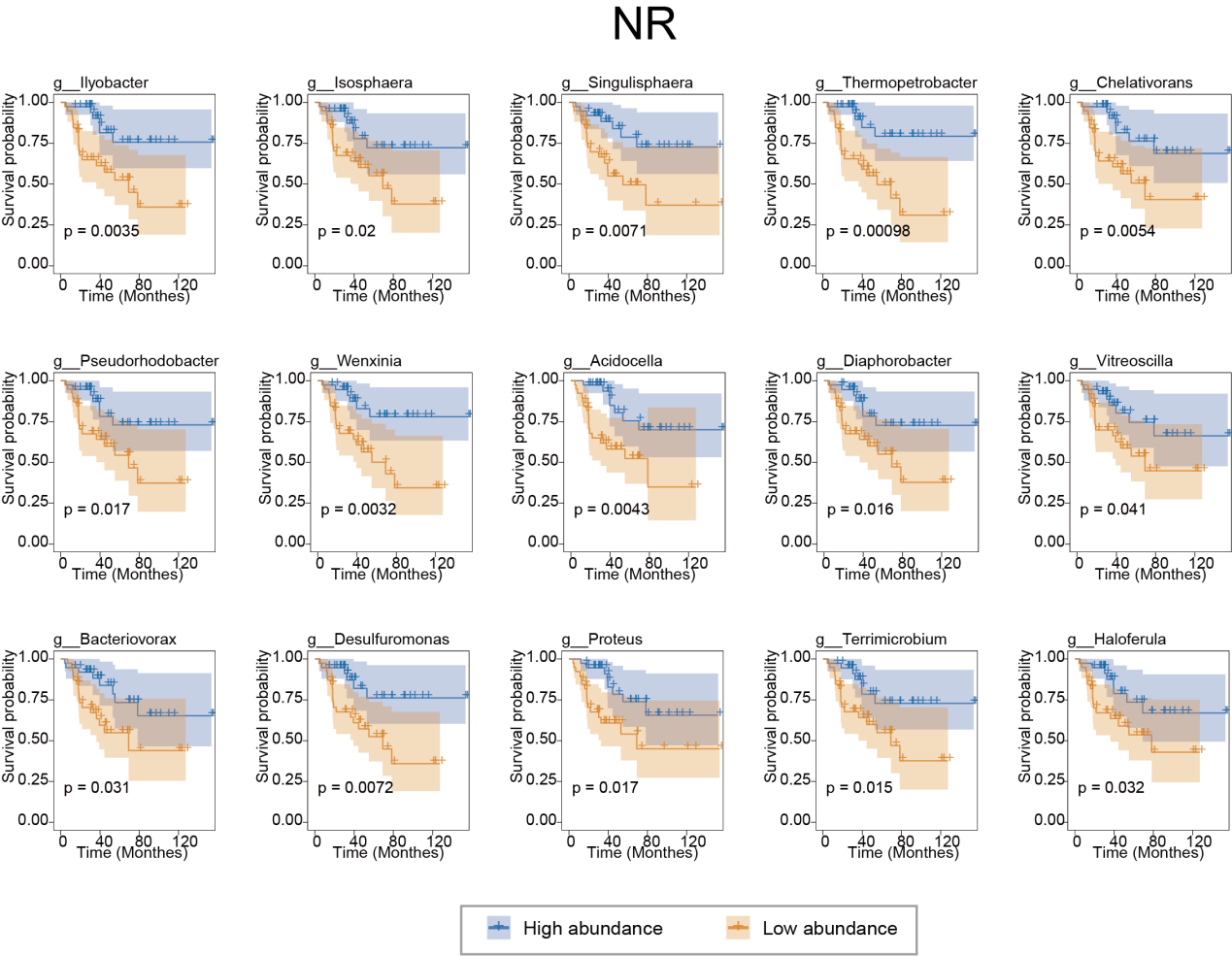


Figure S9. Survival distribution based on the abundance levels of 15 microbial signatures in NR data. The 77 ACC patients were stratified into two groups (high abundance vs. low abundance) based on the median abundance of one of 15 genus signatures in NR data. The Log-rank test gave the p value. NR: no contaminants removed.


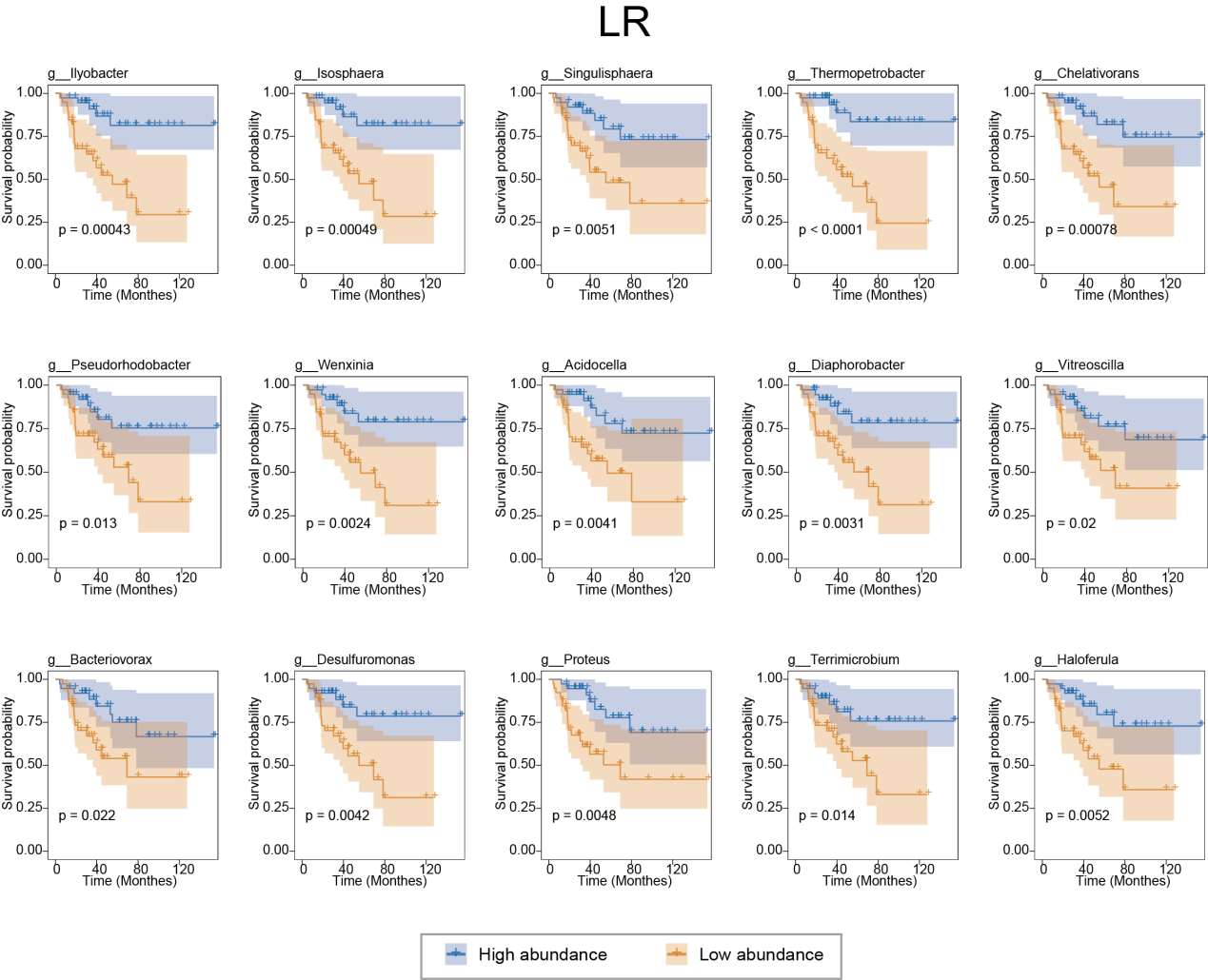


Figure S10. Survival distribution based on the abundance levels of 15 microbial signatures in LR data. The 77 ACC patients were stratified into two groups (high abundance vs. low abundance) based on the median abundance of one of 15 genus signatures in LR data. The Log-rank test gave the p value. LR: Likely contaminants removed.


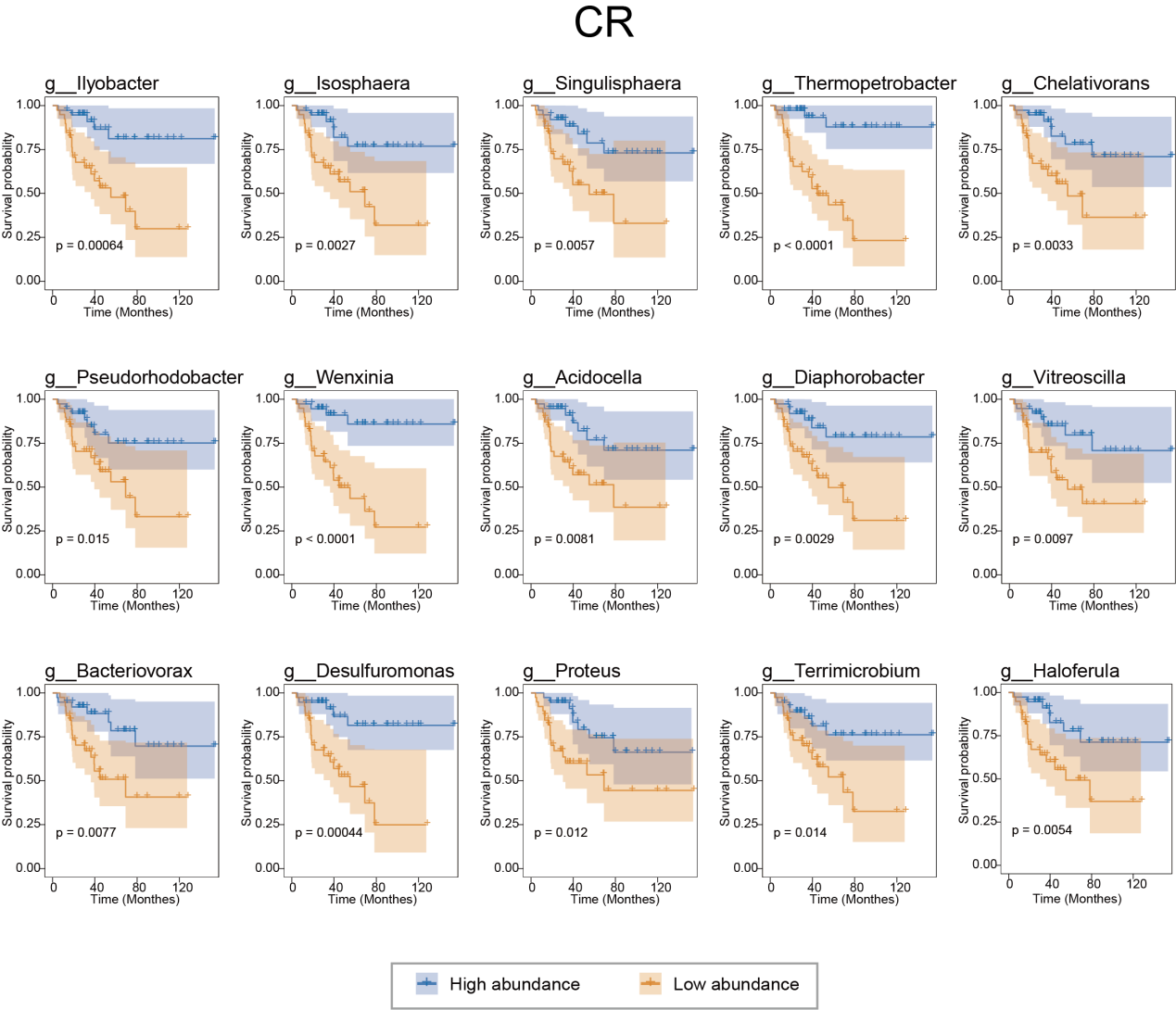


Figure S11. Survival distribution based on the abundance levels of 15 microbial signatures in CR data. The 77 ACC patients were stratified into two groups (high abundance vs. low abundance) based on the median abundance of one of 15 genus signatures in CR data. The Log-rank test gave the p value. CR: contaminants removed by sequencing ‘Plate-Center’ combinations.


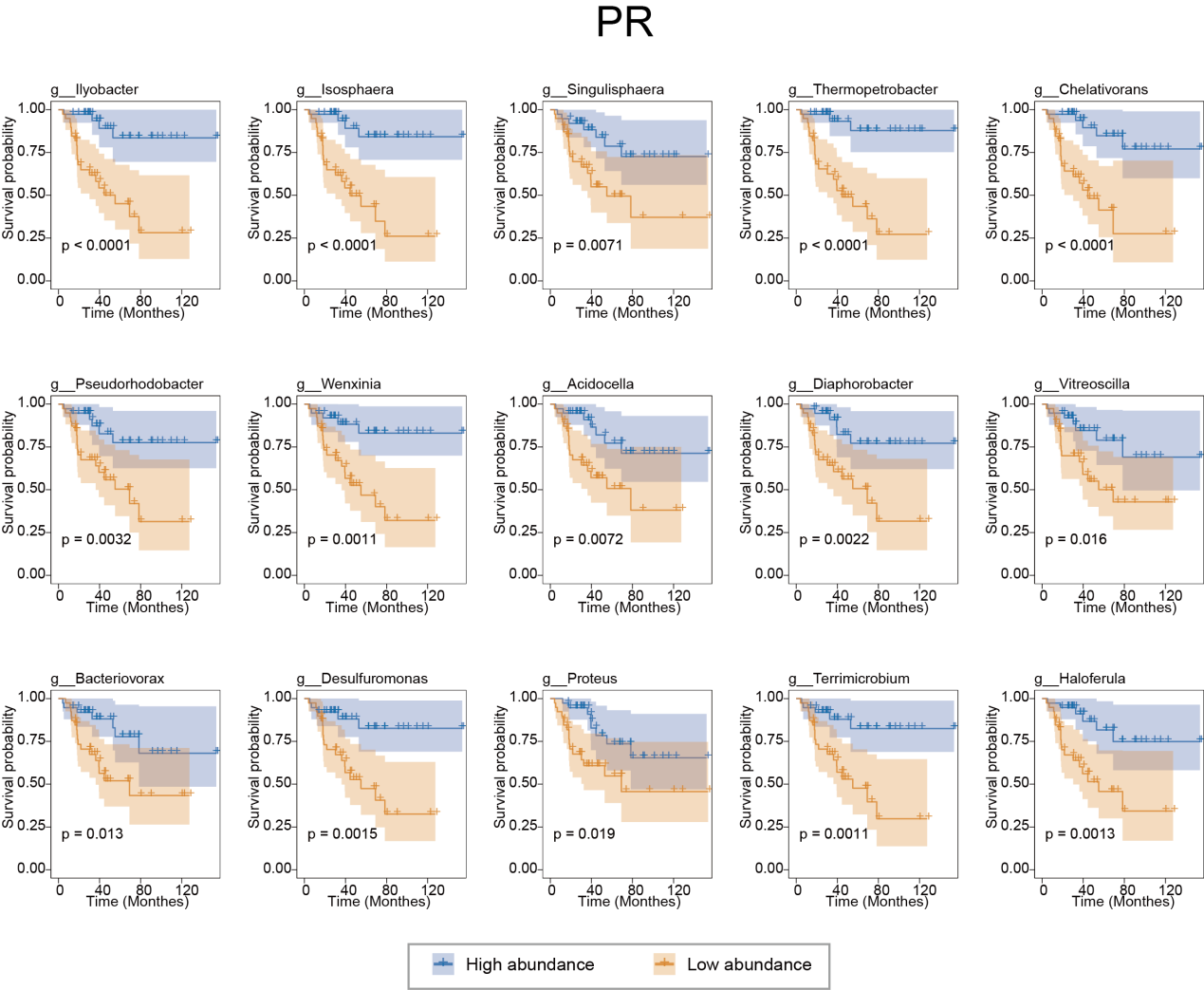


Figure S12. Survival distribution based on the abundance levels of 15 microbial signatures in PR data. The 77 ACC patients were stratified into two groups (high abundance vs. low abundance) based on the median abundance of one of 15 genus signatures in PR data. The Log-rank test gave the p value. PR: all putative contaminants removed.


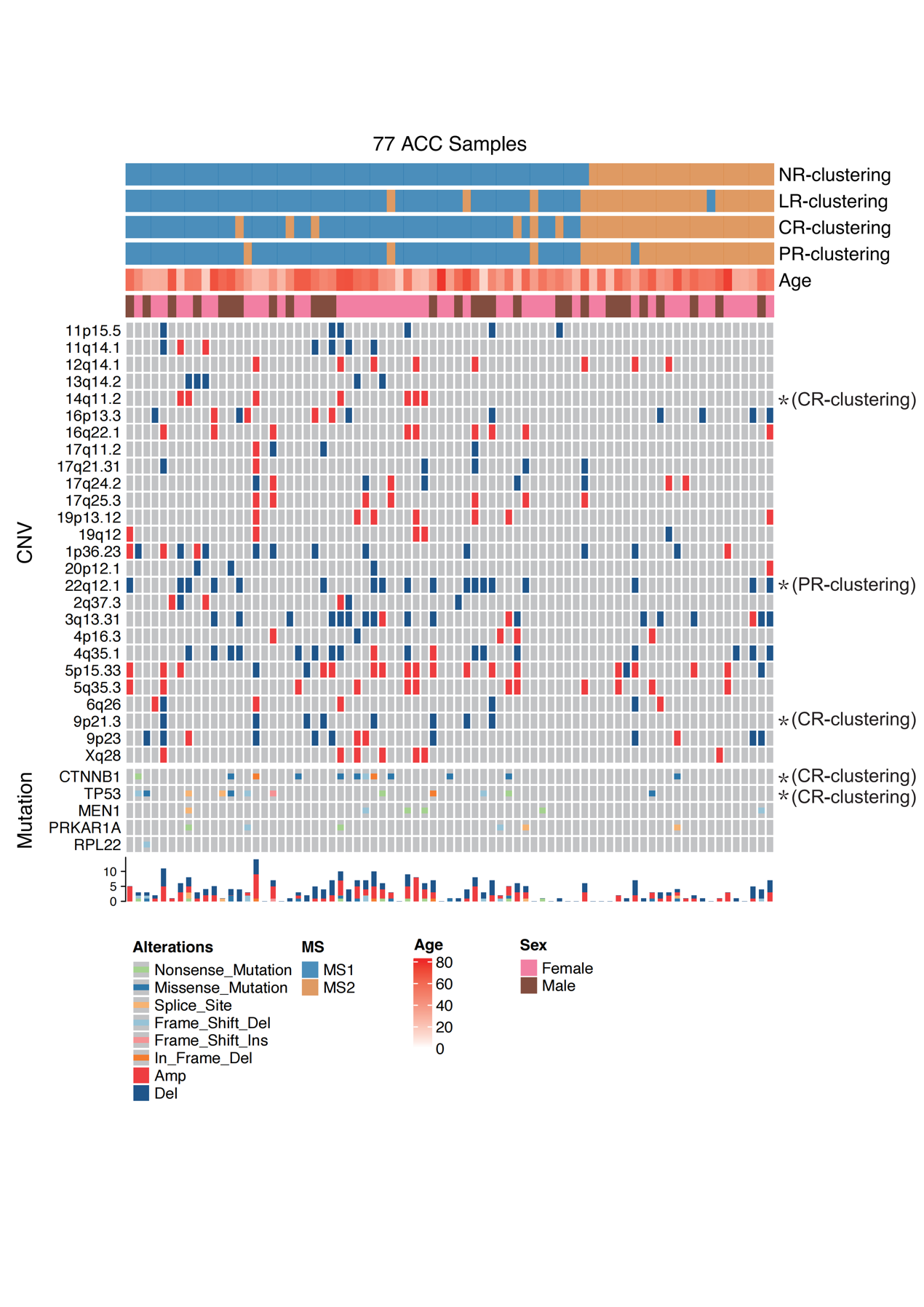


Figure S13. Landscape of copy number variations (CNVs) and driver mutations between two clusters (MS1 & MS2) in ACC. Reproduced from TCGA ACC dataset, shown was waterfall plot of copy number variations (CNVs) and driver mutations in ACC categorized by MS clustering. Genomic event frequencies were compared between two cohorts and the significant different events in were marked on the right of the plot. The significance was shown with asterisks. **P* < 0.05; ***P* < 0.01; ****P* < 0.001.

Figure S14. The p53 pathway-related genomic events were enriched in MS1. Shown were the pathways enriched by the genes with frequent mutational and fragmental events in MS1 cohort. Bar plot showing fraction of pathway affected and fraction of samples affected by genomic alterations in MS1 cohort in (**A**)NR, (**B**)LR, (**C**)CR, (**D**)PR clustering.

**
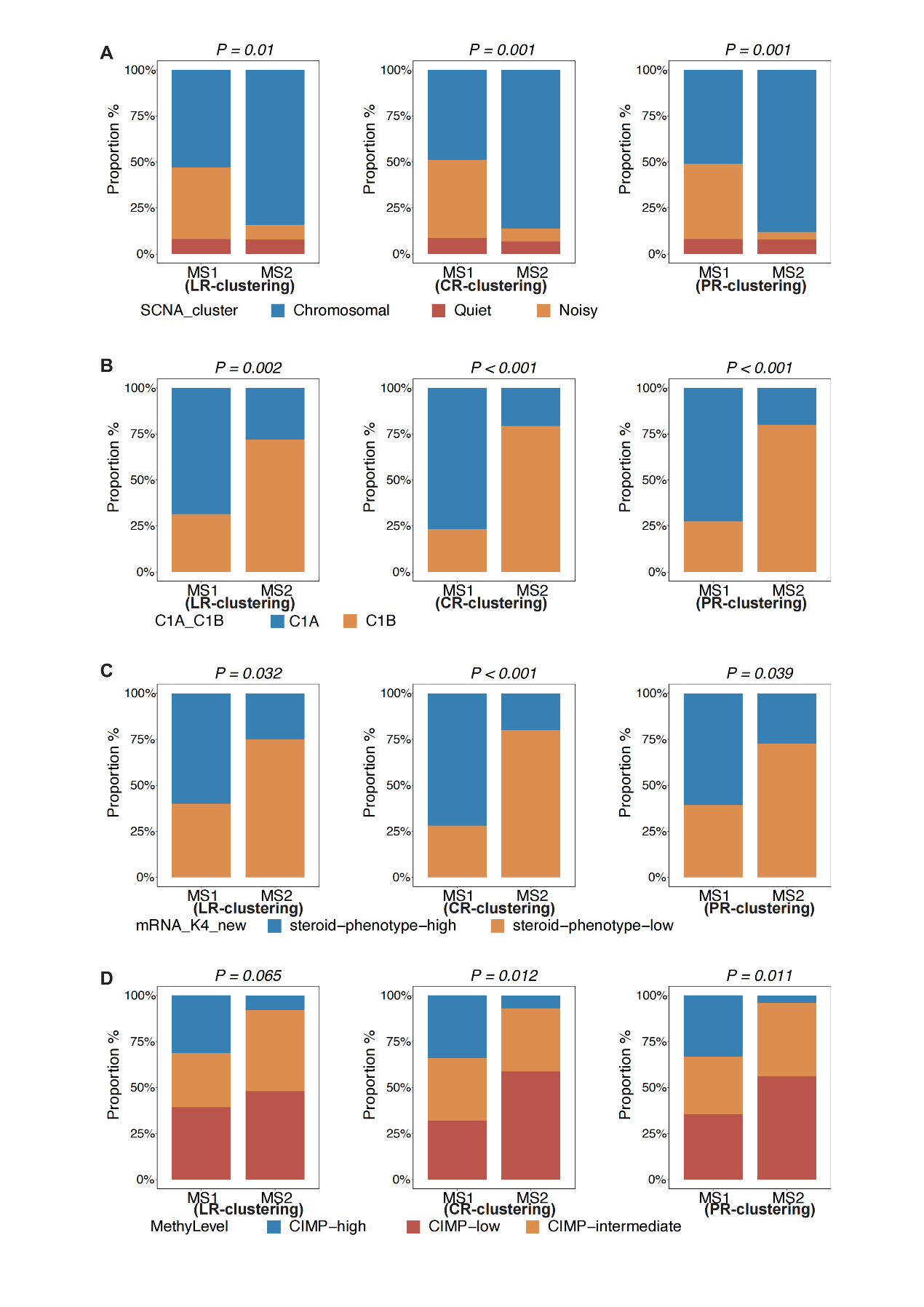
**

Figure S15. The correlation between MS and clinical parameters (significant in four clusterings). Stacked bar plot showing chi-squared test of (**A**) somatic copy number alteration (SCNA) cluster, (**B**) C1A/C1B, (**C**) mRNA cluster, (**D**) methylation cluster between MS1 and MS2 in LR (left panel), CR (middle panel) and PR (right panel) clustering.


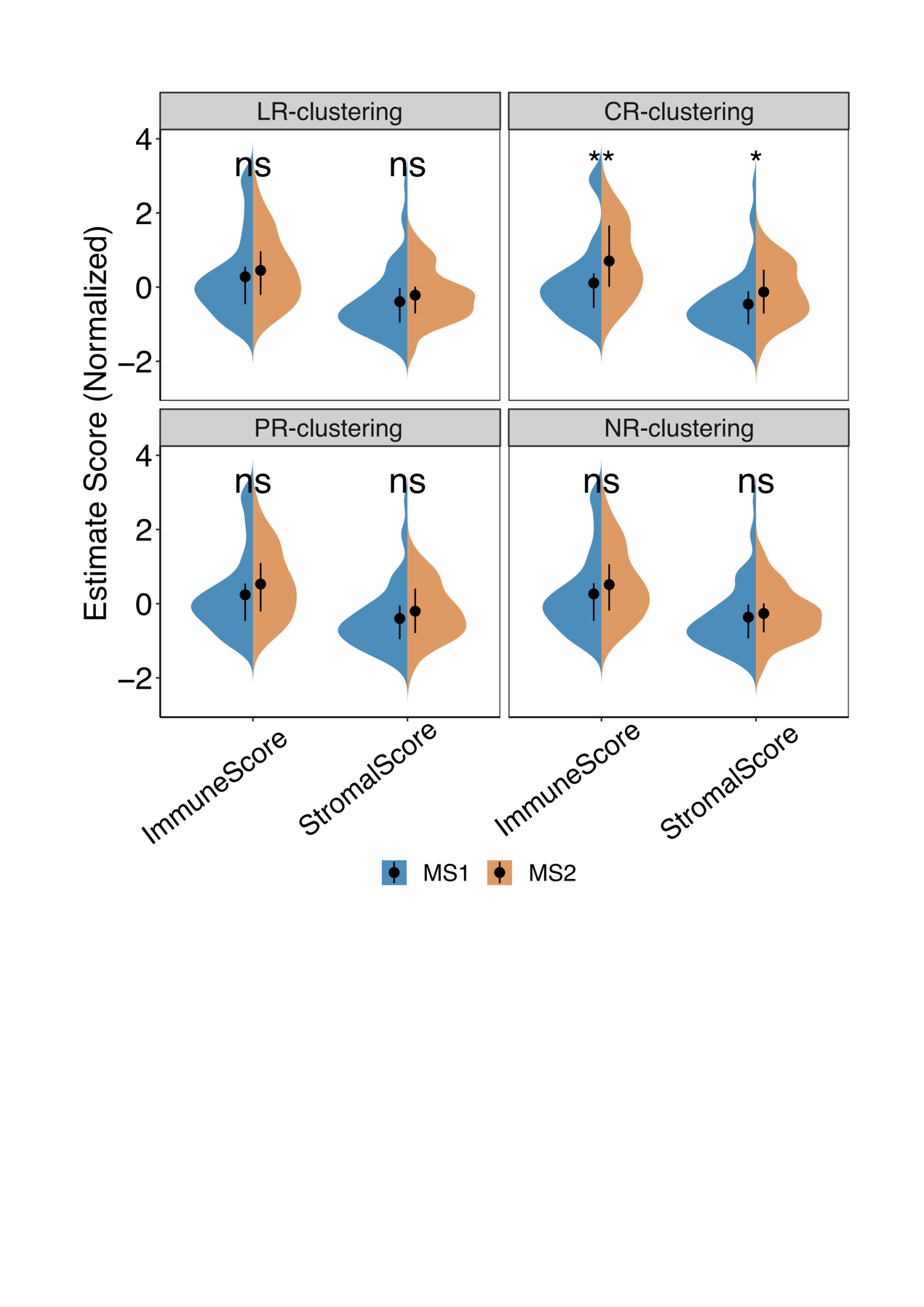


Figure S16. Immune estimation score between two clusters. Violin plot showing difference of immune score and stromal score between MS1 and MS2 in four clusterings. The significance was shown with asterisks. **P* <0.05; ***P* < 0.01; ****P* < 0.001; ns, not significant.

**
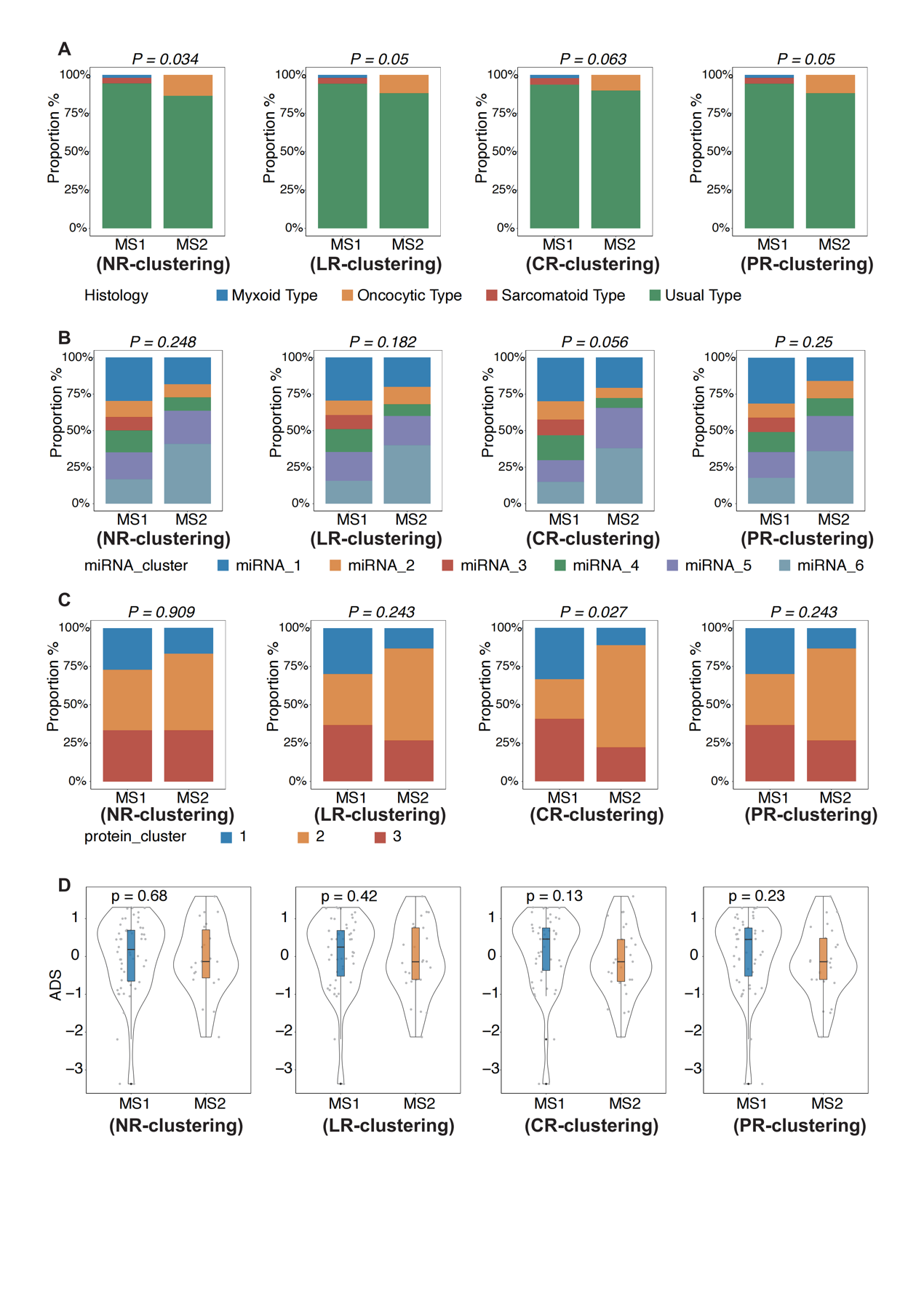
**

Figure S17. The correlation between MS and clinical parameters (not significant in four clusterings). Stacked bar plot showing chi-squared test of (**A**) histology cluster, (**B**) miRNA cluster, (**C**) protein cluster, (**D**) adrenocortical differentiation score (ADS) between MS1 and MS2 in NR, LR, CR and PR clustering.

Figure S18. Links between gut microbial taxa and host genes in cell cycle pathway and p53 signaling pathway. **(A)** Procrustes analysis showing overall association between variation in host gene expression and gut microbiome composition in ACC. We used Bray-Curtis dissimilarity for host gene expression data (triangles) and microbiome data (circles). (**B**) Links between microbial taxa and host genes in the cell cycle and p53 signaling pathway. The size of circles and triangles represents the absolute value of sparseCCA coefficients of genes and microbes, respectively.
